# Early inflammation precedes cardiac fibrosis and heart failure in desmoglein 2 murine model of arrhythmogenic cardiomyopathy

**DOI:** 10.1007/s00441-021-03488-7

**Published:** 2021-07-08

**Authors:** K. E. Ng, P. J. Delaney, D. Thenet, S. Murtough, C. M. Webb, N. Zaman, E. Tsisanova, G. Mastroianni, S. L. M. Walker, J. D. Westaby, D. J. Pennington, R. Pink, D. P. Kelsell, A. Tinker

**Affiliations:** 1 William Harvey Research Institute, Barts and The London School of Medicine and Dentistry, Queen Mary University of London, Charterhouse Square, London, EC1M 6BQ UK; 2 Blizard Institute, Barts and The London School of Medicine and Dentistry, Queen Mary University of London, London, E1 2AT UK; 3 School of Biological and Chemical Sciences, Queen Mary University of London, Mile End Road, London, E1 4NS UK; 4 CRY Dept. of Cardiovascular Pathology, Cardiology Clinical Academic Group, Molecular and Clinical Sciences Research Institute, St. George’s University of London, Jenner WingCranmer Terrace, London, SW17 0RE UK; 5 Department of Biological and Medical Sciences, Faculty of Health and Life Sciences, Oxford Brookes University, Headington Campus, Oxford, OX3 0BP UK

**Keywords:** Desmoglein 2 (*Dsg2*), Arrhythmogenic cardiomyopathy (AC), Desmosome, Cardiac inflammation and macrophages

## Abstract

The study of a desmoglein 2 murine model of arrhythmogenic cardiomyopathy revealed cardiac inflammation as a key early event leading to fibrosis. Arrhythmogenic cardiomyopathy (AC) is an inherited heart muscle disorder leading to ventricular arrhythmias and heart failure due to abnormalities in the cardiac desmosome. We examined how loss of desmoglein 2 (*Dsg2*) in the young murine heart leads to development of AC. Apoptosis was an early cellular phenotype, and RNA sequencing analysis revealed early activation of inflammatory-associated pathways in *Dsg2*-null (*Dsg2*^*−/−*^) hearts at postnatal day 14 (2 weeks) that were absent in the fibrotic heart of adult mice (10 weeks). This included upregulation of iRhom2/ADAM17 and its associated pro-inflammatory cytokines and receptors such as TNFα, IL6R and IL-6. Furthermore, genes linked to specific macrophage populations were also upregulated. This suggests cardiomyocyte stress triggers an early immune response to clear apoptotic cells allowing tissue remodelling later on in the fibrotic heart. Our analysis at the early disease stage suggests cardiac inflammation is an important response and may be one of the mechanisms responsible for AC disease progression.

**Supplementary Information:**

The online version contains supplementary material available at 10.1007/s00441-021-03488-7.

## Introduction

Arrhythmogenic cardiomyopathy (AC), also referred to as arrhythmogenic right ventricular cardiomyopathy (ARVC), is an inherited heart muscle disease characterised by ventricular arrhythmias, notably ventricular tachycardia and fibrillation, and later in the disease process heart failure (Austin, et al. 2019; Sen-Chowdhry et al. 2010). It is often a cause of cardiac arrest in young athletes. The condition appears to have acute arrhythmogenic phases before a decline in ventricular function that occurs later in the disease. Pathologically, it is characterised by cardiac chamber dilatation and fibrofatty replacement of myocytes. Historically, it was thought to be a predominantly a right ventricular disease, but biventricular and left ventricular patterns are now widely recognised (Austin et al. 2019; Sen-Chowdhry et al. 2010). The disease is hereditary in origin occurring in about 1 in 5000 births and is generally autosomal dominant though recessive forms are recognised. Both may present with prominent extracardiac features involving the skin (palmoplantar keratoderma) and/or woolly hair. The genetic basis for the disease was first revealed by the study of autosomal recessive forms of the cardiocutaneous form of AC and demonstrated loss-of-function mutations in desmoplakin (DSP) (Carvajal syndrome) and plakoglobin (JUP) (Naxos disease) (McKoy et al. 2000; Norgett et al. 2000). Both of these genes encode proteins that are part of the desmosome. This prompted studies in autosomal dominant AC including the examination of other desmosomal genes and revealed mutations in other components of the cardiac desmosome including desmoglein (DSG2), desmocollin2 (DSC2) and plakophilin2 (PKP2) (Syrris et al. 2007, 2006a, b). Desmosomes tether cardiomyocytes together by linking cells at the intercalated disc with the intermediate filaments, specifically desmin, in the cytoskeleton (Vermij et al. 2017). Together with gap junctions and adherens junctions in the area composita of the intercalated disc, they provide mechanical and electrical connection between cells (Kowalczyk and Green 2013).

AC can be a largely asymptomatic dormant condition until the initial cardiac episode occurs in later life. These disease triggers underlying AC are still largely unknown though exercise and/or infection are proposed external factors with desmosomal dysregulation and cell adhesion as likely consequences. Early observations from myocarditis patient biopsies previously suggested the involvement of a systemic viral trigger; however, no research has been able to confirm this as the cause (Calabrese et al. 2000, 2006; Campuzano et al. 2012; Nishikawa et al. 1999). Interestingly, subsequent studies described the presence of inflammatory infiltrates in postmortem biopsies from patients with AC (Campuzano et al. 2012). Interestingly, clinical reports of myocarditis in recent paediatric cases highlight the connection between inflammation and AC (Martins et al. 2018). Human genetic studies have been useful in identifying desmosomal genes that cause the disease; however, difficulties in obtaining human samples and other limitations such as exposures to environmental stressors like exercise can vary between individuals carrying similar desmosomal mutations. Hence, there are now a number of murine models for AC; these transgenic models have been generated to unravel the cellular and molecular onset of disease progression.

One of the first murine models involved cardiac haploinsufficiency of desmoplakin and recapitulated many of the clinical features of AC (Garcia-Gras et al. 2006). (Gomes et al. 2012). This was followed by the development of other mouse models including those involved in the genetic manipulation of *Dsg2* (Eshkind et al. 2002; Kant et al. 2015; Krusche et al. 2011; Pilichou et al. 2006, 2009; Rizzo et al. 2012). The global genetic deletion of *Dsg2* is embryonically lethal whilst heterozygotes with haploinsufficiency appear normal (Eshkind et al. 2002). In contrast, transgenic mice overexpressing a missense *dsg2* mutation or a knock-in mouse lacking extracellular EC1-EC2 adhesive domain of *Dsg2* develops cardiac dysfunction similar to that observed in AC patients (Krusche et al. 2011; Pilichou et al. 2009). The findings from Krushe et al. reported abnormal cardiac morphology and the presence of fibrotic lesions in 2-week-old *Dsg2* mutant mice. The *Dsg2* defect caused structural changes where adult mice exhibited dilated cardiomyopathy and compromised cardiac function. Later studies from the same group confirmed the presence of inflammatory infiltrate from 4-week-old *Dsg2* mutant mice (Kant et al. 2015). New interest in this field now supports the notion that inflammation does indeed shape disease pathogenesis in murine AC (Lubos et al. 2020). The data gathered from the various *Dsg2* mouse models to date and our own study highlights the importance of cardiac inflammation in response to defective desmosomes and how we can use this knowledge to understand AC disease progression in humans.

Much of the recent focus is on the benefits of using pharmacological agents to effectively target inflammatory pathways creating new therapies for AC (Chelko et al. 2016, 2019). In our study, we examine the transgenic murine model with cardiac-specific deletion of *Dsg2*. The basis for our work was to provide further mechanistic insight into cardiac inflammation in the early stages of heart development. We identify an early inflammatory response with unique gene expression patterns and propose key events that may occur in the postnatal murine heart and how these signatures can mould disease phenotype later on in the fibrotic adult heart.

## Materials and methods

### Generation of genetically modified mice

Animals were cared for according to the Animals (Scientific Procedures) Act 1986. Mice were maintained in an animal core facility under UK Home Office guidelines related to animal welfare. Mice were kept in individually ventilated, pathogen-free, temperature-controlled cages (21–23 °C) with 12-h day/night light cycles and free access to standard rodent chow and water. The *Dsg2* tm1c allele was generated at MRC Harwell (Oxfordshire, UK). Our study mice were generated by crossing mice expressing the cardiac αMHC-Cre promoter (alpha-myosin heavy chain promoter; *Myh6*) with *Dsg2* floxed mice where exon 4–5 of the desmoglein 2 gene is flanked by *loxP* sites. This mating resulted in Cre-mediated excision of desmoglein 2 in cardiac myocytes, resulting in a frameshift mutation with premature truncation. Breeding pairs were set up to generate wild-type *Dsg2*^+/+^ (Cre^−^, *Dsg2*^flx/flx^) and knockout *Dsg2*^−/−^ mice (Cre^+^, *Dsg2*^flx/flx^). Mice from both sexes were studied at 2, 6 and 10 weeks of age to characterise the histology, inflammatory and stress pathways involved and electrophysiological properties of the failing hearts. Ear samples were taken at postnatal day 13 to identify the genotype of the 2-week cohorts, and pups were sacrificed the next day for additional analyses.

### In vivo assessment of cardiac function

In vivo cardiac assessment was performed with a VisualSonics (VisualSonics Inc., Toronto, Canada) Vevo 3100 high-resolution ultrasound scanner with a 30-MHz frequency transducer. Mice were anaesthetised with 1.5% (v/v) isoflurane whilst the body temperature was maintained at 37 °C with a temperature probe. B- and M-mode parasternal long-axis and short-axis views were acquired to evaluate cardiovascular structures and cardiac function. Doppler scans were also taken to determine blood flow velocity and direction, as evidenced by colour differential. Electrocardiograms (ECGs) were also obtained with the built-in ECG electrode contact pads. ECG measurements were processed in LabChart (ADI Instruments, UK), and data was analysed in Prism 8.0. The Vevo LAB 3.1.1 analysis software (VisualSonics Inc., Toronto, Canada) was used for quantifying the images. The most commonly used variables to evaluate systolic function are fractional shortening (FS) and ejection fraction (EF) measured from changes in chamber dimensions during systole (S) and diastole (D). Measurements and calculations were obtained from short-axis M mode with the left ventricle (LV) trace method; results were saved and processed in Excel before statistical analysis in Prism 8.0.

### Electrophysiological mapping

We used a flexible multielectrode array (FlexMEA; Multi Channel Systems, Germany) to study the electrophysiological properties of the intact heart as we have previously described (Aziz et al. 2018; Finlay et al. 2019). The data collected was used to calculate conduction velocity (CV), mean increase in delay (MID) and effective refractory period (ERP) from our study cohort (n = 8, both groups). Hearts were perfused in the Langendorff mode with normal oxygenated Krebs solution (Ca^2+^ 1.4 mM) at 16.5 ml/min. A unipolar silver chloride stimulation electrode and a flexible 32-pole multielectrode array (MEA) (FlexMEA, Multielectrode System, Germany) were placed on the ventricular epicardium, and an S_1_S_2_ decremental protocol was performed to measure the ventricular effective refractory period (VERP). A biphasic pulse of amplitude 2 V and duration 0.5 ms was used for stimulation, with S_1_S_2_ intervals reduced from 150 ms by decrements of 5 to 100 ms followed by decrements of 2 ms until tissue refractoriness was reached. Arrhythmogenicity was further tested for by applying stimulating trains of 100 beats at coupling intervals progressively reduced from 100 ms. Ventricular tachycardia was defined as a ventricular arrhythmia lasting more than 2 s.

All analysis of murine electrophysiology was performed using custom software running in Matlab, v2014b (MathWorks Inc., MA, USA). The time point of local activation was taken at the steepest negative gradient of the unipolar electrogram. Conduction velocities were determined using a gradient method, with conduction velocity defined as the inverse of the gradient in activation times across the array. Electrodes with significant noise were excluded, and all electrograms and time points were checked manually. MID, a well-validated measure of the inducibility of conduction delay, was calculated by determining the area under the conduction-delay curve. The mean timing of the activation time of all recording electrodes was used for each measurement of conduction delay, and the MID defined as the unit increase in conduction delay per unit reduction in S_1_S_2_ coupling interval (ms/ms). Stimulation protocols were performed in normal Krebs–Henseleit solution (Sigma, UK).

### Tissue collection and morphometric analysis

Following the ex vivo whole heart experiments, hearts were rinsed immediately in phosphate-buffered saline (PBS) before they were placed into a tissue holder and cut longitudinally using both right and left atrial appendages as a guideline. Excess moisture from both halves was blotted off before the weights of the individual hearts were recorded. One half was fixed in 10% formalin for 24 h and paraffin-embedded for histology and immunostaining. The other half was cut, and approximately 30 mg of the left ventricle was stored in RNAlater (Sigma, UK). Samples were obtained from 2-week- and 10-week-old mice for qPCR analysis (n = 5, both groups). Whole hearts were collected from a separate cohort of 2-week-old mice for fluorescence-activated cell sorting (FACS) analysis (n = 7, both groups).

### Murine histology and immunohistochemistry

Mouse hearts collected from 2- and 10-week-old animals were rinsed thoroughly in PBS to remove excess blood and fixed in 10% formalin for at least 24 h. After the fixation process, they were washed twice in PBS and stored in 70% ethanol before paraffin embedding. Paraffin-embedded myocardia were cut into 5-μm-thick sections and mounted on clear Plus microscope slides. For histological analysis, sections were stained for haematoxylin and eosin with automated Leica autostainer XL system (Leica Biosystems, UK) and trichrome stain kit (Ab150686, Abcam, UK) to detect cardiac fibrosis (according to the manufacturer’s instructions).

### Electron microscopy

The fixed tissues from 2-week-old mice were cut into 1-mm pieces, post-fixed with 1% osmium tetraoxide and finally embedded in Araldite resin (CY212) kit. Ultrathin sections were post-stained with uranyl acetate and lead citrate and examined with a transmission electron microscope, JEM 1230 (JEOL), at a voltage of 80 kV and a Morada (EMSIS) CCD camera.

### DAB staining

To identify immune cell populations from 2-week *Dsg2*^−/−^ hearts, we employed the chromogenic tissue-staining method using the DAB detection system. The automated Ventana Classic and XT system (Roche Diagnostics, UK) was used to process our paraffin-embedded heart samples. The antibodies used were rat monoclonal CD45 (Ab25386, Abcam, UK) and rat monoclonal F4/80 (MCA497GA Clone CI:A3-1, Serotec, UK). The OmniMap anti-rabbit HRP kit (760–4311, Roche Diagnostics, UK) was used to detect the antibodies of interest. The samples were counterstained with haematoxylin; slides were sealed with mountant and coverslip and allowed to dry. Sections from *Dsg2*^+/+^ and *Dsg2*^−/−^ hearts were also stained with *Dsg2* antibody (Ab150372, Abcam, UK) to confirm that *Dsg2* had been deleted in the heart. Slides from the various histological stains were scanned with a Pannoramic 250 high-throughput scanner (HistoTech, Budapest, Hungary), and representative images from 2-week and 10-week samples with scale bars were processed with the Panoramic Viewer software (HistoTech, Budapest, Hungary).

### Immunofluorescent staining

Samples from paraffin-embedded 2-week- and 10-week-old mouse hearts were dewaxed followed by antigen retrieval with citrate buffer pH 6.0 (H-3300, Vector Laboratories, UK) for 10 min in the microwave. Slides were then rinsed in PBS and permeabilised with 0.25% Triton in PBS for 15 min. Slides were washed several times in PBS and blocked in 5% goat serum (GS) in PBS at room temperature (RT) for 1 h. The samples were incubated overnight in 1% GS in PBS at 4 °C with the following antibodies: desmin (M0760, clone D33, Dako), n-cadherin (H-63) (Sc-7939, Santa Cruz), collagen 1 antibody Col1A1 and troponin T (Ab21286 and ab8295, Abcam, UK). The slides were washed in PBS and incubated for 1 h at RT with 2 μg/ml of the following secondary antibodies: 488 goat anti-mouse, 555 donkey anti-rabbit or 555 goat anti-mouse (A-31572, A-11011 and A-11004, Invitrogen, UK). The samples were washed with PBS and counterstained with DAPI for 5 min before the slides were mounted a 22 mm × 50 mm coverslip with Immunomount (Shandon, UK). The slides were kept in the dark and imaged with confocal microscopy (Zeiss LSM 710, Carl Zeiss, UK). The images were processed in ImageJ.

### TUNEL staining

Paraffin samples from 2-week-old mice were stained for terminal deoxynucleotidyl transferase–mediated dUTP nick-end labelling (TUNEL) assay to confirm apoptosis caused by DNA fragmentation. The ApopTag® Plus In Situ Apoptosis Fluorescein Detection Kit (S7111 Sigma-Aldrich, UK) was used following the manufacturer’s instructions. The slides were kept in the dark and imaged with confocal microscopy (Zeiss LSM 880 with Airyscan Fast, Carl Zeiss, UK). The number of TUNEL-positive cells and total number of cell nuclei counterstained with DAPI were calculated in ImageJ with ITCN plugin (nucleus counter) to generate the % of TUNEL-positive cells (n = 3 both groups).

### Western blotting

Heart tissue was harvested and washed with PBS. The tissue was cut up and placed in lysis buffer containing T-Per Tissue Protein Extraction Reagent (Thermo Fisher, UK) and EDTA-free Complete Protease Inhibitor tablet (Roche, UK). The tissue was then homogenised in the lysis buffer using the Precellys Evolution tissue homogeniser (Bertin Instruments) at 0 °C for 3 × 20 s bursts. After centrifugation for 10 min at 4 °C, the supernatant containing protein was removed. The protein concentration was measured using a Bradford assay kit (Bio-Rad, UK). Equal amounts of protein were loaded and separated by sodium dodecyl sulphate–polyacrylamide gel electrophoresis (SDS-PAGE) (on 10% polyacrylamide gels) and transferred to a nitrocellulose membrane (Whatman). The blots were incubated with antibodies against *Dsg2*, *Adam17*, alpha-tubulin and GAPDH (Ab150372, ab2051, ab2461 and ab9485, Abcam, UK). Blots were developed according to the manufacturer’s instructions (ECL Immobilon Western, Millipore).

### RNA sequencing analysis

RNA samples from 2- and 10-week hearts (n = 4, both groups) were assessed for quantity and integrity using a NanoDrop 8000 V2.0 spectrophotometer (Thermo Scientific, USA) and Agilent 2100 Bioanalyser (Agilent Technologies, Waldbronn, Germany). All samples displayed low levels of degradation with RNA integrity numbers (RIN) between 7.6 and 9.1. One hundred nanograms of total RNA from each sample was used to prepare total RNA libraries using the KAPA Stranded RNA-Seq Kit with RiboErase (KAPA Biosystems, Massachusetts, USA). Fragmentation prior to first-strand cDNA synthesis was carried out using incubation conditions recommended by the manufacturer for intact RNA samples (94 °C for 6 min), and 14 cycles of PCR were performed for final library amplification. Resulting libraries were quantified using a Qubit 2.0 spectrophotometer (Life Technologies, California, USA), and the average fragment size was assessed using the Agilent 2200 TapeStation (Agilent Technologies, Waldbronn, Germany). Equimolar amounts of each sample library were pooled together, and 75-bp paired-end reads were generated for each library using the Illumina NextSeq® 500 high-output sequencer (Illumina Inc., Cambridge, UK).

In Galaxy v19.05, FASTq files were mapped to the GRCm38.97 mouse ensemble genome using RNAstar v2.6, gene and transcript counts were made with StringTie v1.3.4 and GTF merged tables of all samples were made by StringTie merge v1.3.4, recounted using StringTie and differential expression tested by edgeR v3.24.1/Deseq2 v1.18.1. KEGG pathway analyses were generated using DAVID Bioinformatics Resources, version 6.8 (https://david.ncifcrf.gov/home.jsp) (Huang 2009a, b). Gene lists for analysis were identified using a cut-off of adjusted *p*-value < 0.05 and log2 fold-change ≥ 1 and ≤  − 1. KEGG outputs were filtered using false discovery rate (FDR) *p*-value < 0.05, and data was log10 transformed for visualisation. Immune signatures were selected for the 2-week samples. The heat map was generated from 41 differentially expressed immune-related genes. An adapted version (including several macrophage-related genes) of the ‘Mouse nCounter**®** Immunology Panel’ (nanoString) was used to filter genes, and differentially expressed genes were defined as adjusted *p*-value < 0.05, log2 fold-change ≥ 1.5 and ≤  − 1.5, base mean > 500. Heat map was plotted using the pheatmap package (v1.0.12) in R (v3.6.2). RNA sequencing (RNA-Seq) data are presented with FPKM (fragments per kilobase of transcript per million mapped reads) values. The data discussed in this publication have been deposited in NCBI’s Gene Expression Omnibus (Edgar et al. 2002) and are accessible through GEO Series accession number GSE153124 (https://www.ncbi.nlm.nih.gov/geo/query/acc.cgi?acc=GSE153124).

### Quantitative real-time PCR

Total RNA (from ~ 30 mg of tissue) was extracted from 2- and 10-week mouse heart samples stored in RNAlater with the RNeasy fibrous tissue kit (74,704, Qiagen, UK). RNA was quantified using a NanoDrop spectrophotometer (Thermo Fisher Scientific, UK), and total RNA was DNase I treated and reverse-transcribed using a high-capacity cDNA reverse transcription kit (4,368,813, Applied Biosystems, Life Technologies, UK). Fifty nanograms of cDNA was used for quantitative real-time PCR (qRT-PCR), which was performed using customised Taqman gene expression assays (Applied Biosystems, UK). Commercially available probes for genes of interest were used; see Table 1. Each gene was assayed in triplicate, and relative expression was calculated by using the comparative CT method normalised to GAPDH (n = 5 for both groups and time points). The data are presented as relative changes when compared with the control group (*Dsg2*^+/+^) at 2 weeks (set at one arbitrary unit).

**Table 1:** List of mouse probes used in this study. All probes were obtained from Thermo Fisher Scientific, UK

Gene	Catalogue ID number (FAM)
*Dsg2*	Mm00514608_m1
*Col1A1*	Mm00801666_g1
*Col3A1*	Mm01254476_m1
*Ctgf*	Mm01192933_g1
*Tgfβ2*	Mm00436955_m1
*Il-6*	Mm00446190_m1
*Ptprc*	Mm01293577_m1
*Rhbdf2*	Mm00553470_m1
*Adam17*	Mm00456428_m1
*Ccr2*	Mm99999051_gH
*Ccl2*	Mm00441242_m1

### Fluorescence-activated cell sorting

Mice were sacrificed by neck dislocation; hearts were harvested and rinsed in PBS. The tissue was cut into small pieces and then processed in digestion buffer containing Hanks’ medium (10-527F, Lonza, UK), 608 U/ml of collagenase I (C9891m, Sigma, UK), 187.5 U/ml collagenase XI (C7657, Sigma, UK), 90 U/ml hyaluronidase (H3884, Sigma, UK) and 90 U/ml DNAse (D4263, Sigma, UK). Samples were incubated at 37 °C in a shaking incubator at 100 rpm for 1.5 h. The contents were then passed through 70-µm cell strainers to remove debris, and the filtrate was diluted with PBS. These were centrifuged to create a pellet, and the cells were resuspended at a concentration of 2 × 10^6^ cells/50 µl. Fc blocking was performed using TruStain FcX™ CD16/32 antibody (anti-mouse) to avoid non-specific binding (101,320, BioLegend, UK) according to the supplier protocols The fluorochrome-conjugated antibodies used in the panel were obtained from BioLegend, UK: CD11c-BV605 (117,333), CD8-AF488 (100,723), Ly6G-PE (127,607), F4/80-PE/Cy5 (123,111), CD11b-PE/Cy7 (101,215), CD4-APC (100,412) and CD45.2-AF700 (109,822). The antibodies Ly6C-eF450 (48–5932-80) and CD3-PerCP/eF710 (46–0032-80) were obtained from eBioscience, UK. Fluorescence Minus One (FMO) controls were included for each marker. The samples were analysed using an LSRFortessa system (BD Biosciences, UK) equipped with the BD FACSDiva™ software (BD Biosciences, UK), followed by analysis using FlowJo (Version 10.2, Treestar, Ashland, OR). The CD45 + counts were normalised to the total number of events run; the final data show the percentage of the different cell types that were CD45 + .

### Statistical analysis

All results are presented as mean ± SEM where n is the number of mice used. Statistical analyses were conducted using GraphPad Prism (version 8.0; GraphPad Software, California, USA). For comparison of two sets of data, a two-tailed, unpaired Student’s t-test was used. For the analysis of three or more groups, one-way ANOVA followed by Dunnett’s or Tukey’s multiple comparisons tests were used where p < 0.05 was statistically significant.

## Results

Mice harbouring the tm1c allele for the *Dsg2* gene (Supporting Fig. 1A) were crossed with the alpha MHC Cre mice (Agah et al. 1997) and then bred to a second generation to give the study cohort: Cre^+^, *Dsg2*^flx/flx^ (*Dsg2*^*−/−*^*, Dsg2* knockout KO) or control *Dsg2*^flx/flx^ (*Dsg2*^+*/*+^*, Dsg2* wild-type WT) mice. Newborn mice appear normal, and there are no striking physical differences between the adult *Dsg2*^+*/*+^ and *Dsg2*^*−/−*^ mice; the results from morphometric analysis showed no differences between the two groups (unpublished data). We also generated a small cohort of *Dsg2* heterozygous mice (*Dsg2*^+/−^) to examine cardiac function and the effect of gene dosage in comparison to human AC disease development (Pilichou et al. 2006). We show desmoglein 2 was deleted in the heart in this novel cardiac-specific mouse line. DAB staining, RNA-Seq, qPCR and Western blot analysis confirmed substantial decreases in transcripts translated into protein for *Dsg2* (Supporting Fig. 1B, C).

**Fig. 1:**
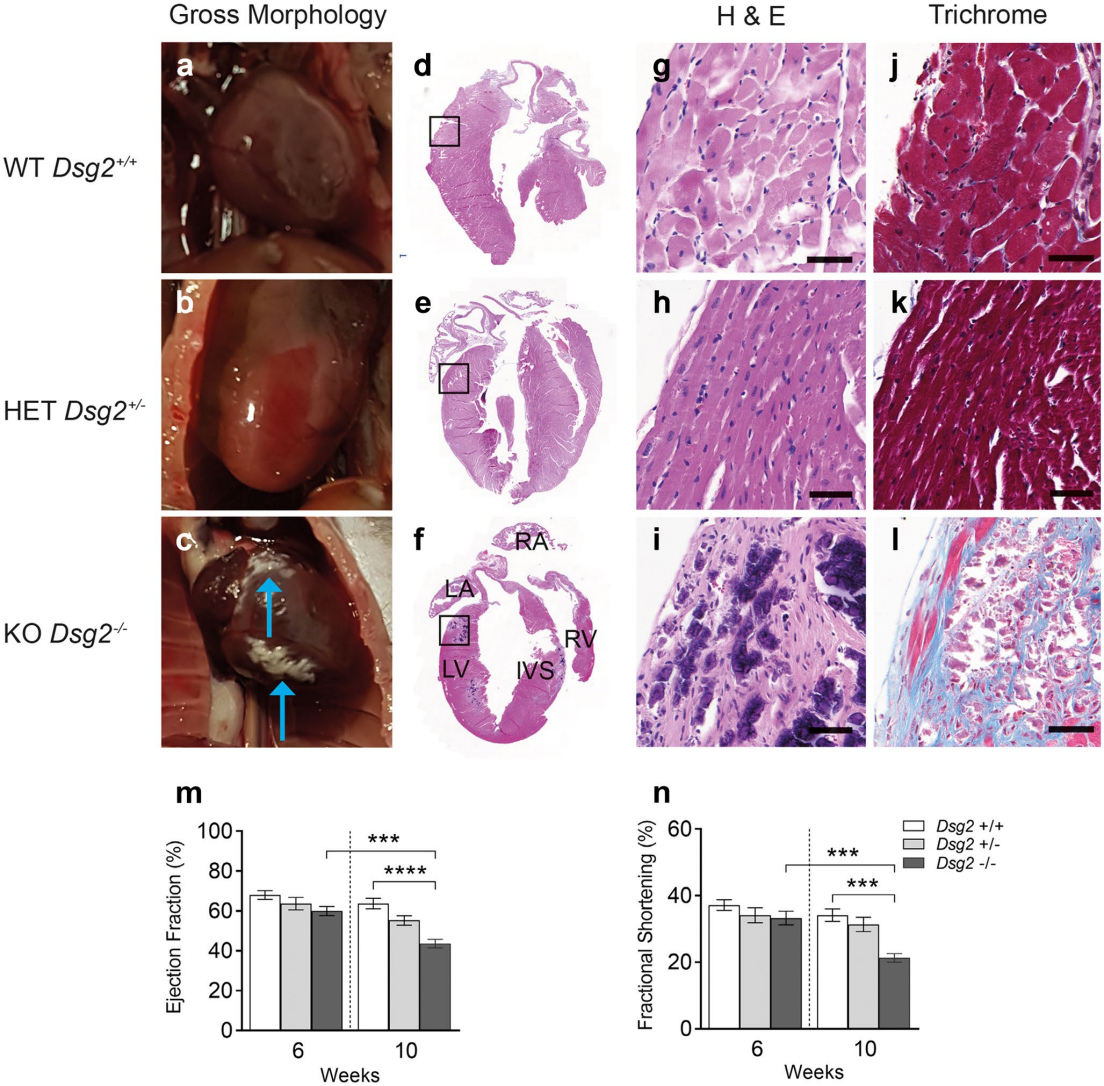
Histological and cardiac assessment of cardiac-specific desmoglein 2 mouse model. The *Dsg2*^*−/−*^ adult mouse exhibits severe cardiac dysfunction due to gross morphological defects. (**a**–**l**) Detailed histology of whole hearts at 10 weeks with zoomed-in areas on the epicardial surface using H&E and Masson’s trichrome stain in wild-type (WT) *Dsg2*^+/+^ (**a**, **d**, **g** and **j**), heterozygous (HET) *Dsg2*^+*/−*^ (**b**, **e**, **h** and **k**) and knockout (KO) *Dsg2*^−/−^ (**c**, **f**, **i**, **l**) hearts. The *Dsg2*^+/+^ (**a**) and *Dsg2*^+*/−*^ (**b**) hearts visually appear normal. However, the extreme phenotype in the *Dsg2*^−/−^ heart (**c**) shows the myocardium with white fibrous plaques. These plaques have extensive calcinosis, which appears as purple deposits (**f**, **i**). These areas also stain positive (blue) for collagen (**l**). Scale bar in g–l represents 50 µm. Abbreviations on *Dsg2*^*−/−*^ heart (**f**) are as follows: LV, left ventricle; RV, right ventricle; LA; left atrium; RA, right atrium; and IVS, intraventricular septum. (**m, n**) Cardiac assessments of this mouse model showed ejection fraction (m) and fractional shortening (n) were significantly reduced between *Dsg2*^+*/*+^ and *Dsg2*^−/−^ hearts by 10 weeks (*Dsg2*^+/+^ and *Dsg2*^+/−^ n = 8 and *Dsg2*^−/−^ n = 7). There is also a significant decline in cardiac function in *Dsg2*^*−/−*^ heart in mice from 6 to 10 weeks. All graphs represent mean ± SEM. ***p < 0.001 and ****p < 0.0001

### Adult Dsg2^−/−^ hearts exhibit fibrosis, whereas adult Dsg2^+/+^ and Dsg2^+/−^ hearts appear morphologically similar

We examined the heart in this murine model for signs of AC by comparing three groups: *Dsg2* WT (*Dsg2*^+*/*+^), heterozygous HET (*Dsg2*^+*/−*^) and knockout KO (*Dsg2*^*−/−*^) mice at 10 weeks (Fig. 1). The morphological analysis of the separated hearts shows different phenotypes (a–c). No abnormal morphological changes or fibrotic lesions were observed in *Dsg2*^+*/*+^ (n = 31) (a) and *Dsg2*^+*/−*^ hearts (n = 19) (b), whereas the *Dsg2*^*−/−*^ hearts displayed distinct white fibrotic lesions (c). These epicardial lesions of varying sizes were present on the left, right or sometimes both ventricles in all adult *Dsg2*-deficient mice (n = 30). Further pathological examination of dissected hearts showed ~ 50% of *Dsg2*^*−/−*^ hearts also had similar lesions within the intraventricular septum or apex. Anatomically, dilation of the LV was visible in the majority of subjects (90%) with a small proportion of individuals exhibiting a dilated right ventricle or enlarged atria (15%). The degree of LV cardiodilation in the *Dsg2*^*−/−*^ heart is evident in the short-axis mode (Supporting Fig. 1D). Most cardiac *Dsg2*^−/−^ mice died within 6 months. Histological analysis with haematoxylin and eosin (H&E) and Masson’s trichrome stain revealed gross cardiac pathological differences between *Dsg2*^+*/*+^ (d, g and j) and *Dsg2*^*−/−*^ hearts (f, i and l). The *Dsg2*^*−/−*^ example shown in c shows epicardial and subepicardial fibrosis (arrows) with similar lesional areas within the septum. The morphological changes observed in the *Dsg2*^−/−^ heart is consistent with replacement fibrosis. Other distinct histological features include collagen deposits and in severe cases calcification (~ 50% of the *Dsg2*-deficient mice), which appear as dark purple aggregates (i) in advanced lesions as reported by earlier studies (Kant et al. 2015). Additional histological examples of 10-week-old *Dsg2*^*−/−*^ hearts are shown in Supporting Fig. 2. Example 1 shows extensive epicardial to midwall fibrosis of the left ventricle with patchy epicardial fibrosis of the right ventricle. Occasional lesions are present in the septum and subendocardium. Example 2 shows biventricular epicardial fibrosis with areas of transmural fibrosis. A subendocardial lesion is present in the left ventricle.

**Fig. 2:**
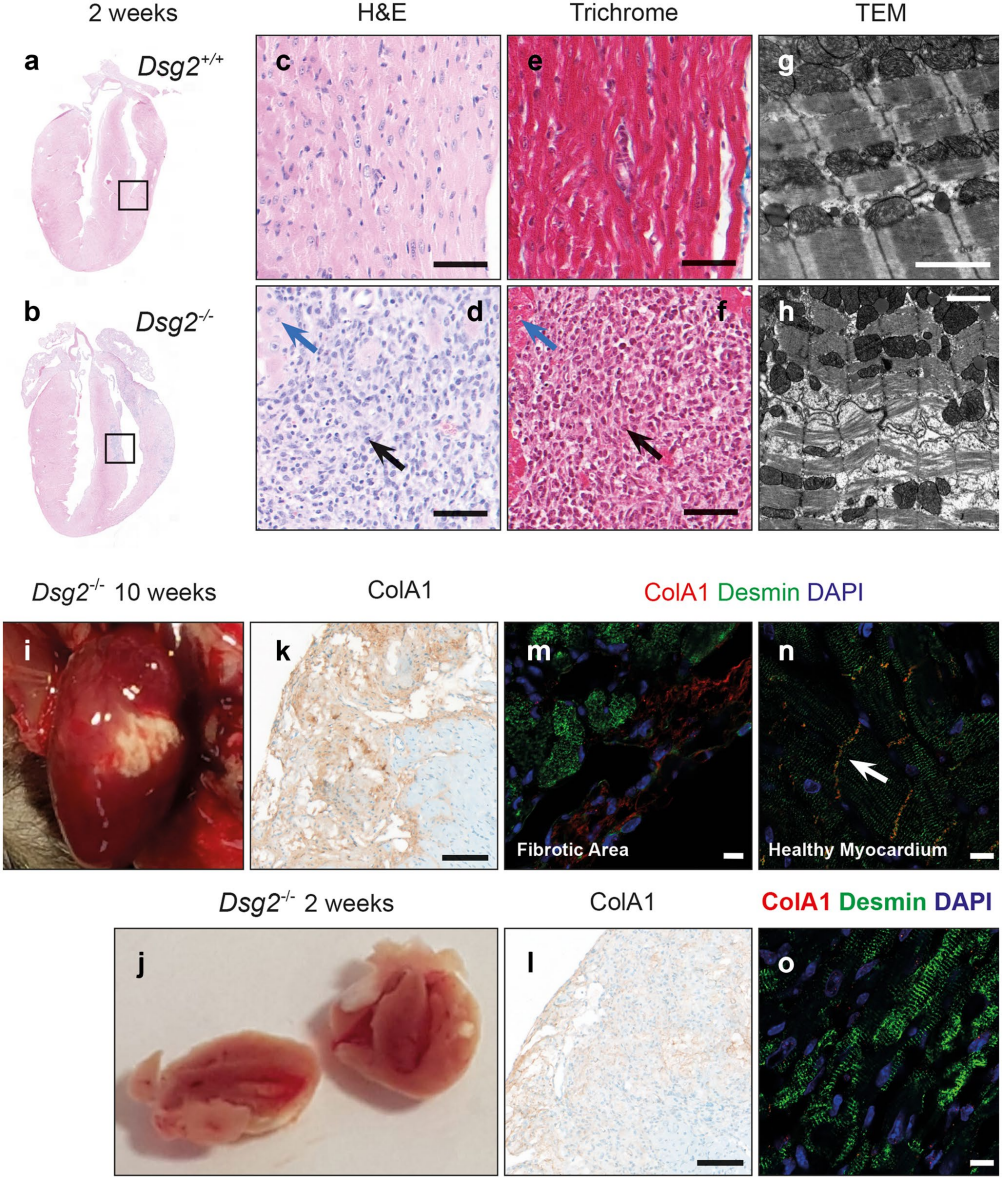
Gross morphological changes are evident in *Dsg2*^−/−^ hearts at 2 weeks of age. Histological analysis confirms early signs of inflammation. (**a–h**) Detailed analysis of *Dsg2*^+/+^ and *Dsg2*^−/−^ hearts with H&E, Masson’s trichrome stain and electron microscopy. (**a**) The myocardium in *Dsg2*^+/+^ heart appears normal with no abnormal morphology (**c**, **e**). The H&E analysis for the postnatal *Dsg2*^−/−^ heart (**b**, **d**) presents extreme phenotype and inflammation with the accumulation of dense nuclei, predominantly immune cells (black arrow) and some normal cardiomyocytes (blue arrow). (**f**) The 2-week *Dsg2*^−/−^ hearts do not stain positive for collagen, but there are clear changes within the myocardium. Scale bars represent 50 µm (**c**–**f**). (**g**, **h**) Electron micrographs show examples of *Dsg2*^+*/*+^ (**g**) and *Dsg2*^*−/−*^ (**h**) hearts. The *Dsg2*^*−/−*^ sample shows distorted muscle fibres and electron-dense mitochondria, suggesting signs of mitochondrial swelling. Scale bars represent 2000 µm (**g** and **h**). Gross morphological comparison of the adult (**i**) and postnatal *Dsg2*^*−/−*^ (**j**) hearts. Large dense fibrous plaques are present in the adult heart where changes in the extracellular matrix have already taken place, whereas inflammation is the proposed key event in the postnatal heart. (**k**–**o**) Immunohistological analysis reveals collagen deposition is present in the adult *Dsg2*^*−/−*^ heart (**k, m, n**) but not detected in the 2-week *Dsg2*^*−/−*^ heart (**l**, **o**). Desmin is located at the intercalated disc (**n**) when co-stained with n-cadherin in healthy myocardium whilst the lesional area (**m**) shows dysregulation of desmin in the adult *Dsg2*^*−/−*^ heart. Scale bars represent 100 µm (**k** and **l**) and 10 µm (**m**, **n**, **o**)

We measured cardiac function in younger juvenile mice at 6 weeks and took measurements 4 weeks later in adult mice (m and n). The echocardiographic parameters from the three genotypes are shown in Table 2 (*Dsg2*^+*/*+^, *Dsg2*^+*/−*^ n = 8 and *Dsg2*^*−/−*^ n = 7). The heterozygous group was also included in the analysis to reveal if the decline in cardiac function is related to gene dosage. At 6 weeks of age, there are no statistically significant differences in cardiac function between all groups; however, one *Dsg2*^*−/−*^ mouse died between the two time points. The severe morphological changes in the diseased heart at 10 weeks resulted in a substantial decline in EF (m) and in FS (n) when compared to the control *Dsg2*^+*/*+^ group, indicating functional impairment of contractility. The time course and relative decline for cardiac function is shown in Supporting Fig. 3A; the *Dsg2*^−/−^ mice worsen with age where EF and FS significantly decrease by 33% and 39% respectively. Features such as LV dilation observed in *Dsg2*^*−/−*^ hearts are comparable to those found in other *Dsg2* mouse models (Chelko et al. 2016; Krusche et al. 2011).

**Table 2:** Echocardiographic assessments of *Dsg*^+/+^ (WT), *Dsg2*^+/−^ (HET) and *Dsg2*^−/−^ (KO) mice at 6 weeks and 10 weeks of age

Parameter	*Dsg*^+/+^	*Dsg*^+/−^	*Dsg2*^−/−^	*Dsg*^+/+^	*Dsg*^+/−^	*Dsg2*^−/−^
	6 weeks			10 weeks		
HR BPM	504 ± 14.5	515 ± 16	536 ± 20	498 ± 13.3	502 ± 19	516 ± 12
CO ml/min	14.8 ± 1.5	13.85 ± 2.1	15.54 ± 1.73	14.9 ± 2	15.9 ± 2	11.67 ± 1.82
EF %	68 ± 2.23	63.41 ± 3.16	**62.47 ± 2.86** Ϯ	**63.7 ± 2.6** α	58.8 ± 3.25	**43.62 ± 2.18** Ϯ α
FS %	37.18 ± 1.61	34 + 2.25	**33.27 ± 2.05** Ϯ	**34.14 ± 1.88** β	30.8 ± 2.16	**21.28 ± 1.83** Ϯ β
LV mass mg	82.76 ± 10.1	80.63 ± 8.47	90.67 ± 11.1	106.2 ± 9.6	100.3 ± 13.8	114 ± 9.76
IVS, d mm	0.69 ± 0.06	0.68 ± 0.05	0.63 ± 0.07	**0.85 ± 0.05** ¥	0.73 ± 0.05	**0.65 ± 0.03** ¥
IVS, s mm	1.02 ± 0.08	0.94 ± 0.06	0.93 ± 0.12	1.06 ± 0.09	1.05 ± 0.08	0.84 ± 0.06
LVAW, d mm	0.74 ± 0.07	0.75 ± 0.05	0.73 ± 0.08	0.8 ± 0.06	0.76 ± 0.04	0.72 ± 0.04
LVAW, s mm	1.17 ± 0.07	1.13 ± 0.06	1.08 ± 0.11	**1.16 ± 0.06** γ	1.07 ± 0.07	**0.93 ± 0.05** γ
LVID, d mm	3.28 ± 0.13	3.32 ± 0.13	3.42 ± 0.12	3.34 ± 0.15	3.59 ± 0.18	3.73 ± 0.18
LVID, s mm	2.2 ± 0.14	2.35 ± 0.16	2.31 ± 0.16	**2.36 ± 0.14** γ	2.63 ± 0.22	**2.94 ± 0.17** γ
LVPW, d mm	0.77 ± 0.03	0.81 ± 0.06	0.86 ± 0.07	1.02 ± 0.08	0.82 ± 0.06	0.95 ± 0.06
LVPW, s mm	1.08 ± 0.04	1.03 ± 0.04	1.21 ± 0.1	1.24 ± 0.08	1.08 ± 0.05	1.26 ± 0.1

*Dsg2*^+*/*+^ and *Dsg2*^+*/−*^ (n = 8) and *Dsg2*^*−/−*^ (n = 7). Data are presented as the mean ± SEM. Parameters with significant differences have been highlighted in bold. Please refer to Fig. 1 and Supplementary Fig. 2A. Cardiac function significantly declines with age in the *Dsg2*^*−/−*^ group ( From 6 to 10 weeks): Ϯ *** p < 0.001 for both EF and FS. There are also significant differences between *Dsg2*^+*/*+^ (WT) and *Dsg2*^*−/−*^ (KO) groups:  α **** *p* < 0.0001 for EF. β *** *p* < 0.001 for FS. **γ** * *p* < 0.05 for LVAW; d and LVID; s. ¥ ** *p *< 0.01 for IVS; d

*HR* heart rate, *CO* cardiac output, *EF* ejection fraction, *FS* fractional shortening, *LV mass* left ventricular mass, *IVS* interventricular septum, *LVAW* left ventricle anterior wall, *LVID* left ventricle interior diameter, *LVPW* left ventricle posterior wall, *d* diastole, *s* systole

**Fig. 3:**
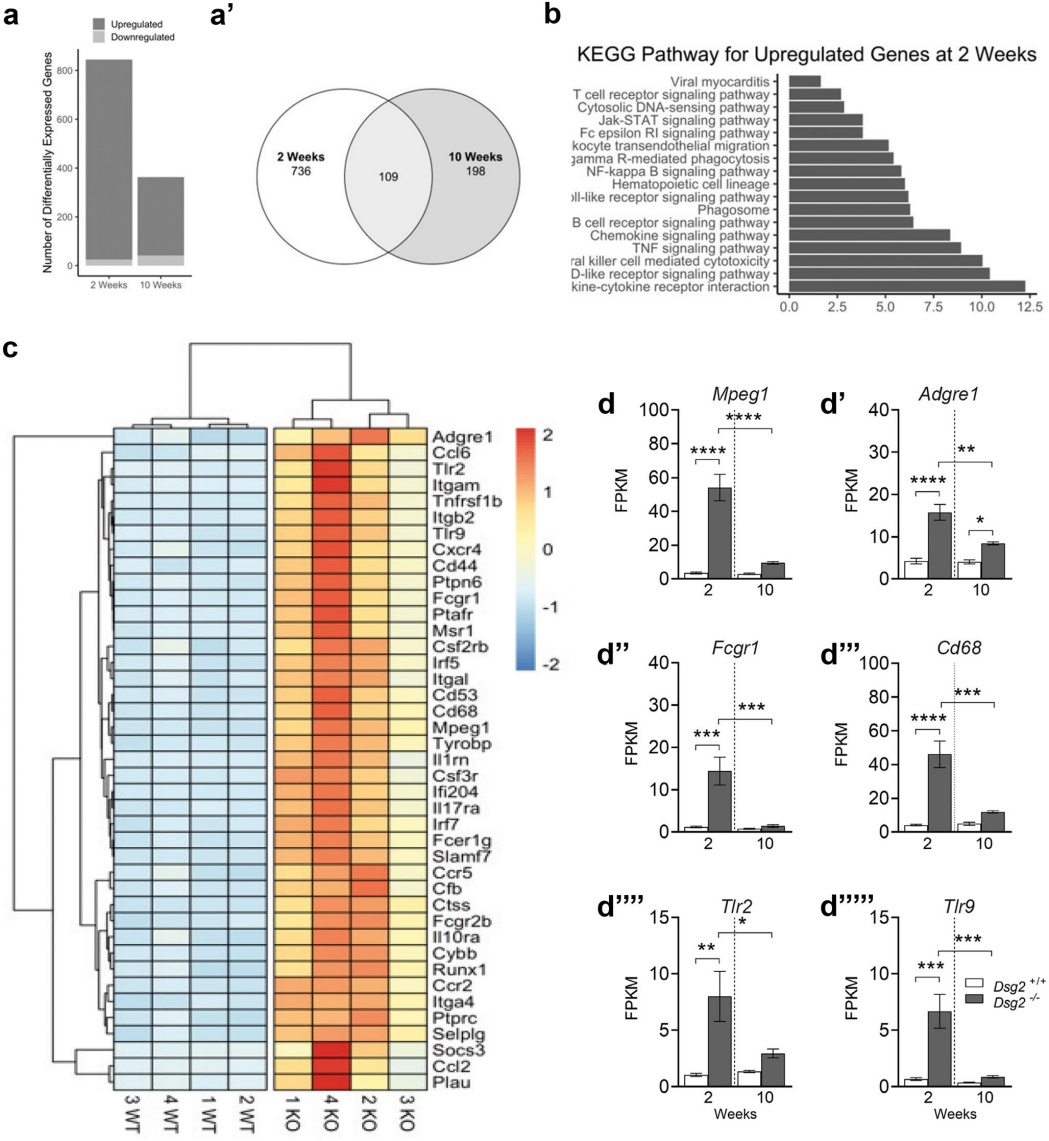
RNA-Seq reveals specific immune cell populations such as macrophages are upregulated in *Dsg2*^−/−^ hearts at 2 weeks of age. (**a**) Bar plot depicting the number of differentially expressed genes, both upregulated and downregulated, between *Dsg2*^+*/*+^ and *Dsg2*^*−/−*^ mice at 2 and 10 weeks (*Dsg2*^+*/*+^ and *Dsg2*^*−/−*^ n = 4, for both time points). (**a′**) The Venn diagram represents the number of differentially expressed genes between *Dsg2*^+*/*+^ and *Dsg2*^*−/−*^ groups at the two time points and the number of genes which cross over between both groups. (**b**) KEGG pathway analyses for upregulated genes at 2 weeks. (**c**) A heat map was generated from 41 differentially expressed immune-related genes. Red for high expression, yellow for medium and blue for low expression of genes. (**d**–**d′′′′′**) Transcriptome data (FPKM = fragments per kilobase of transcript per million mapped reads) show macrophages are the main inflammatory cell population present in *Dsg2*^*−/−*^ hearts. Genes of interest include *Adgre1* (F480) and *Mpeg1* (**d**, **d′**), both highly expressed in mature macrophages. *Fcgr1* and *Cd68* (**d′′**, **d′′′**) are both expressed in monocytes and macrophages, and *Tlr2* and *Tlr9* (**d′′′′**, **d′′′′′**) trigger the innate immune response. Graphs represent mean ± SEM. *p < 0.05, **p < 0.01, ***p < 0.001 and ****p < 0.0001

We examined the electrophysiological properties of the heart using ECG traces from cardiac echocardiographic assessments acquired at 10 weeks. The ECG parameters of interest were compared between *Dsg2*^+*/*+^ and *Dsg2*^*−/−*^ cohorts. Heart rates were normal (beats per minute (BPM) and RR interval). There were no differences with PR interval, P wave duration and QRS interval; however, there was a significant increase in QT and QTc intervals, in the *Dsg2*^−/−^ mice (Supporting Fig. 3B). We further investigated the electrophysiological substrate in Langendorff-perfused isolated hearts using FlexMEA array as previously described (Aziz et al. 2018; Finlay et al. 2019). S_1_S_2_ decremental protocols were performed, and conduction velocity; mean increase in delay, a sensitive measure of conduction delay; and effective refractory period were measured. Capture was, however, inconsistent and challenging with *Dsg2*^−/−^ hearts, as the fibrotic plaques were difficult to avoid; however, there were no significant changes in these parameters (Supporting Fig. 3C).

### Loss of cardiac Dsg2 at 2 weeks present with signs of inflammation

Our studies at 10 weeks show a highly fibrotic and failing heart. The question arises as to what are the initial disease processes that promote this pathological state. To address this, we characterised mice histologically at 2 weeks of age in Fig. 2a–h. The majority of morphological observations from 2-week *Dsg2*^−/−^ hearts (b) showed distinctive small surface lesions (n = 15). There were no abnormal areas in 2-week *Dsg2*^+/+^ hearts (a) (n = 17). The lesions in the *Dsg2*^*−/−*^ cohort were located on the left and right epicardial surfaces although a large number (~ 50% of cases) were also located within the intraventricular septum. No lesions were observed at the apex. Detailed histological analysis revealed the lesions contained densely packed nuclei (d and f); these are absent in the *Dsg2*^+/+^ heart (c and e). These pronounced areas of cellular infiltrates (black arrow) surrounded by existing cardiomyocytes (blue arrow) in the H&E (d) and Masson’s trichrome stain (f) suggest inflammation may be an early event in the lesional areas of *Dsg2*-deficient hearts. The electron micrographs of normal myocardium show light and dark bands (contracted muscle) with mitochondria uniformly distributed along the myofibril in a healthy individual (g). However, in the *Dsg2*^−/−^ heart, there is myocardial disarray with signs of muscle degeneration and abnormal mitochondria (h). The electron-dense mitochondria suggest mitochondrial swelling. Examples of other distinctive ultrastructures observed in the 2-week *Dsg2*^*−/−*^ hearts are shown in Supporting Fig. 4. Distorted muscle fibres are a prominent feature; muscle deterioration is visible at the intercalated disc (A). Swollen and translucent mitochondria are observed throughout the *Dsg2*^*−/−*^ heart; mitochondria with atypical cristae and darker outer membranes are also present (B). There are also swollen mitochondria with myelin bodies and few mitochondria with empty matrixes within the distorted muscle compatible with necrosis and apoptosis (C). D shows a series of images to highlight the histological changes within the lesional area. These images suggest the myocardium is replaced by infiltrating fibroblasts and tissue macrophages with secretory granules.

**Fig. 4:**
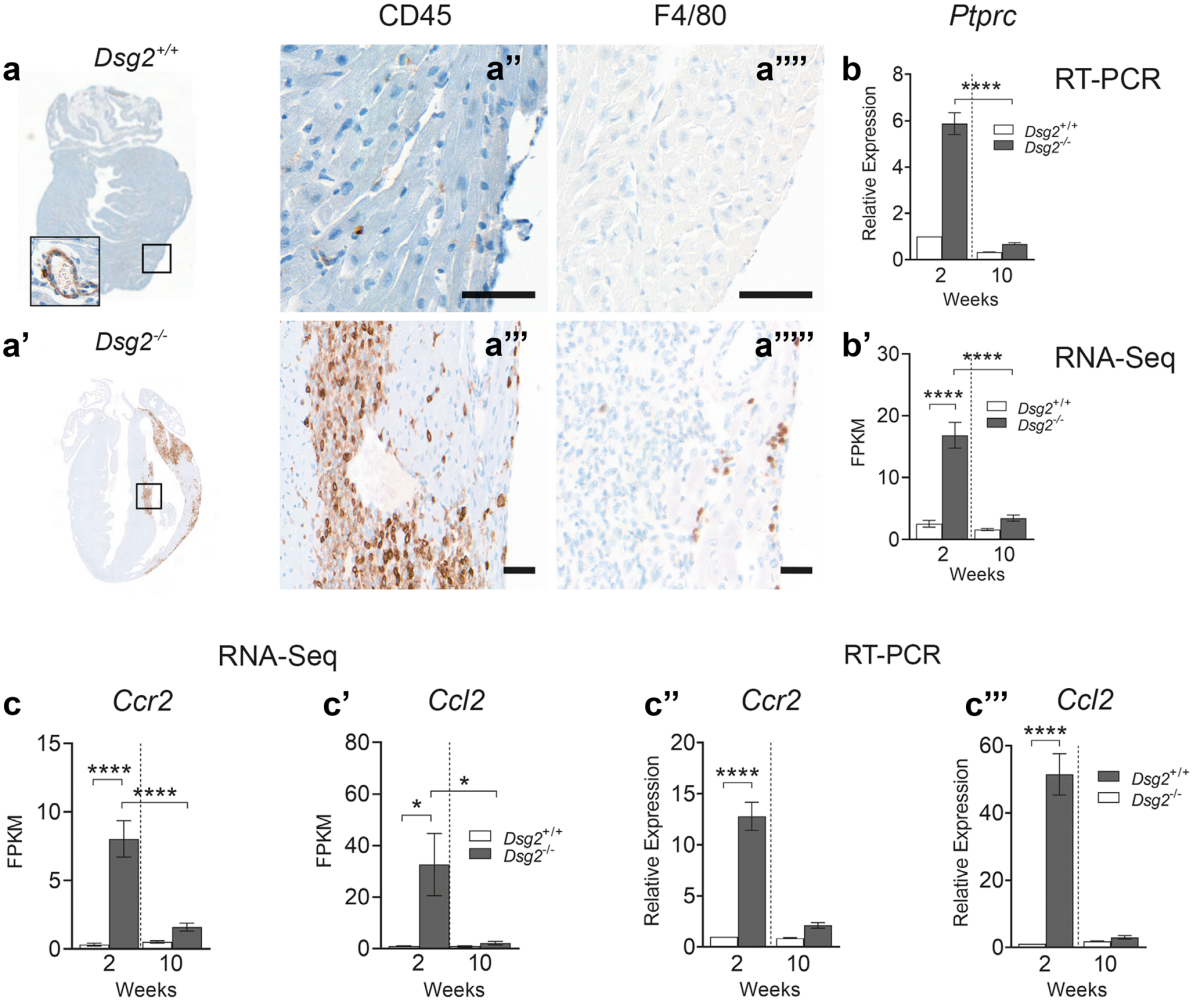
Inflammation and cells from the innate immune response play an important role in the early stages (2 weeks) of desmoglein 2 murine AC disease progression. (**a**–**a′′′′′**) DAB immunostaining analysis in 2-week-old *Dsg2*^+*/*+^ (**a**, **a′′**, **a′′′′**) and *Dsg2*^*−/−*^ (**a′**, **a′′′**, **a′′′′′**) hearts. All scale bars represent 50 µm (**a**–**a′′′′′**). The zoomed-in areas for each heart show the same region where we have stained for CD45 (**a′′**, **a′′′**) and F4/80 (**a′′′′**, **a′′′′′**). There are no immune cells present within the myocardium of the *Dsg2*^+/+^ heart (**a′′**); however, a large population of CD45 + cells are present in the large vessels of the heart. The *Dsg2*^−/−^ heart (**a′′′**) shows increased expression of CD45 + cells on the epicardial surface and within the intraventricular septum region. The selected areas of interest show the presence of macrophages (**a′′′′′**). (**b**, **b′**) To validate the CD45 + DAB results, qRT-PCR (**b**) and RNA-Seq (**b′**) confirmed the presence of CD45 + cells (*Ptprc*) in *Dsg2*^−/−^ hearts at 2 weeks of age when compared to their *Dsg2*^+/+^ littermates. These results confirm that this unique CD45 + signature is only observed in *Dsg2*^*−/−*^ postnatal hearts. (**c**–**c′′′**) Transcriptome data to confirm the activation and recruitment of macrophages in 2-week-old *Dsg2*^−/−^ hearts. *Ccl2* (**c′**, **c′′′**) and its receptor *Ccr2* (**c**, **c′′**) were both highly expressed during this inflammatory stage. All data represent mean ± SEM. *p < 0.05 and ****p < 0.0001

The comparison with the pathology in the 10-week *Dsg2*^−/−^ hearts (i and j) showed the plaque-like lesions were larger and compliant with fibrosis. Immunohistochemistry with an antibody against collagen type 1 confirmed extensive fibrosis in the 10-week *Dsg2*^*−/−*^ heart (k). Desmin is a muscle-specific intermediate filament protein expressed in cardiac, smooth muscle and skeletal muscle. Desmin co-staining showed dysregulation of desmin in stressed or damaged cardiomyocytes that were adjacent to these fibrous plaques in the 10-week *Dsg2*^*−/−*^ heart (m). At higher magnifications in healthy myocardium (n), desmin is located at the intercalated disc (N-cadherin co-staining). There was little or no collagen type 1 detected in the 2-week *Dsg2*^*−/−*^ heart (l). Desmin localises at the Z-discs as the intercalated discs were not visible in our sample (o).

### Comparison of cardiac gene expression at 2 and 10 weeks

We next performed and compared bulk RNA-Seq data from 2- and 10-week-old murine heart tissue to identify potential pathways involved in the early pathogenesis of this disease (Fig. 3). There were a number of significantly differentially expressed genes at 2 weeks when compared to the 10-week dataset (a) with some overlap of common genes between the postnatal and adult periods (a′). The top 15 differentially expressed genes in 2- and 10-week mice are displayed in Tables 3 and 4. The top 15 genes in the 2-week *Dsg2*^*−/−*^ hearts were largely linked to the immune response including *Cd180*, *Card11*, *Trem2* and *Mpeg1*. KEGG pathway analysis between the two time points further indicated that the pathways upregulated at 2 weeks particularly were linked to inflammatory pathways including Janus kinases (JAKs), signal transducer and activator of transcription proteins (STATs), growth factors and pro-inflammatory cytokines such as interleukin 6 (IL-6) (b). In contrast, KEGG pathway analysis at 10 weeks revealed upregulated pathways linked to changes in extracellular matrix and tissue remodelling (focal adhesion and cell adhesion molecules). The dataset also shows that inflammatory pathways (NF-kappa B signalling and cytokine-cytokine receptor) were also active at 10 weeks (Supporting Fig. 5A). It has been well documented that fibrosis is evident in other adult *Dsg2* mouse models (Chelko et al. 2016; Kant et al. 2015; Pilichou et al. 2009). To explore whether fibrotic pathways were altered at 2 weeks in *Dsg2*^*−/−*^ hearts, the expression of several fibrosis-associated genes was assessed. *Col1a1*, *Col3a1* and *Ctgf* were significantly upregulated at 2 weeks in *Dsg2*^−/−^ hearts, although *Tgfβ2* did not change in the early stages of the disease (Supporting Fig. 5B). The real-time PCR data is supported by the findings in Table 3; among the top 15 differentially expressed genes in 2-week *Dsg2*^*−/−*^ hearts include fibrosis-related genes such as *Timp1* (tissue inhibitor of metalloproteinases). In the adult *Dsg2*^*−/−*^heart, *Cola1* and *Col3A1* expression both declined by 10 weeks, *Ctgf* was highly expressed at both time points whilst *Tgfβ2* increased significantly in the adult *Dsg2*^−/−^ heart (Supporting Fig. 5B). Interestingly, *timp-1* is also highly expressed in the adult *Dsg2*^*−/−*^ heart (Table 3).

**Table 3:** Top 15 differentially expressed RNA at 2 weeks. Filtering criteria: log2 fold change ≥ 3 or log2 fold change ≤  − 3 and adjusted p-value < 0.01

Gene name (2 weeks)	Log2 fold change	Adjusted *p*-value
Cd180	3.01740	8.07E − 26
Timp1	3.03955	1.30E − 18
Card11	3.07831	7.62E − 37
Ctss	3.15527	9.79E − 73
Cyp4f18	3.16365	2.83E − 34
Clec4e	3.18540	3.01E − 19
Trem2	3.19016	5.53E − 49
Ly9	3.20628	1.11E − 25
Cd300lb	3.22897	5.46E − 32
Slfn1	3.23281	8.70E − 23
B430306N03Rik	3.29441	7.14E − 24
Gpr35	3.29686	3.31E − 27
Mpeg1	3.38740	2.85E − 53
Adam8	3.48284	1.70E − 37
Serpina3n	3.53029	1.59E − 45

**Table 4:** Top 15 differentially expressed RNA at 10 weeks. Filtering criteria: log2 fold change ≥ 3 or log2 fold change ≤  − 3 and adjusted p-value < 0.01

Gene name (2 weeks)	Log2 fold change	Adjusted *p*-value
Aldob	−2.9868	2.19E − 17
Clcn1	−2.5873	1.19E − 21
Nmrk2	2.0114	1.54E − 14
Ptn	2.0874	6.78E − 12
Fmod	2.0901	3.28E − 19
Angptl7	2.0949	3.10E − 13
Frzb	2.1017	5.77E − 26
P4ha3	2.1726	7.33E − 11
Crlf1	2.2238	1.79E − 12
1500015O10Rik	2.2685	1.38E − 14
Serpinb1c	2.3312	1.51E − 10
Nox4	2.3493	8.68E − 22
Slamf7	2.4634	3.07E − 23
Timp1	2.4924	4.90E − 27
Col8a2	2.4998	6.99E − 21

**Fig. 5:**
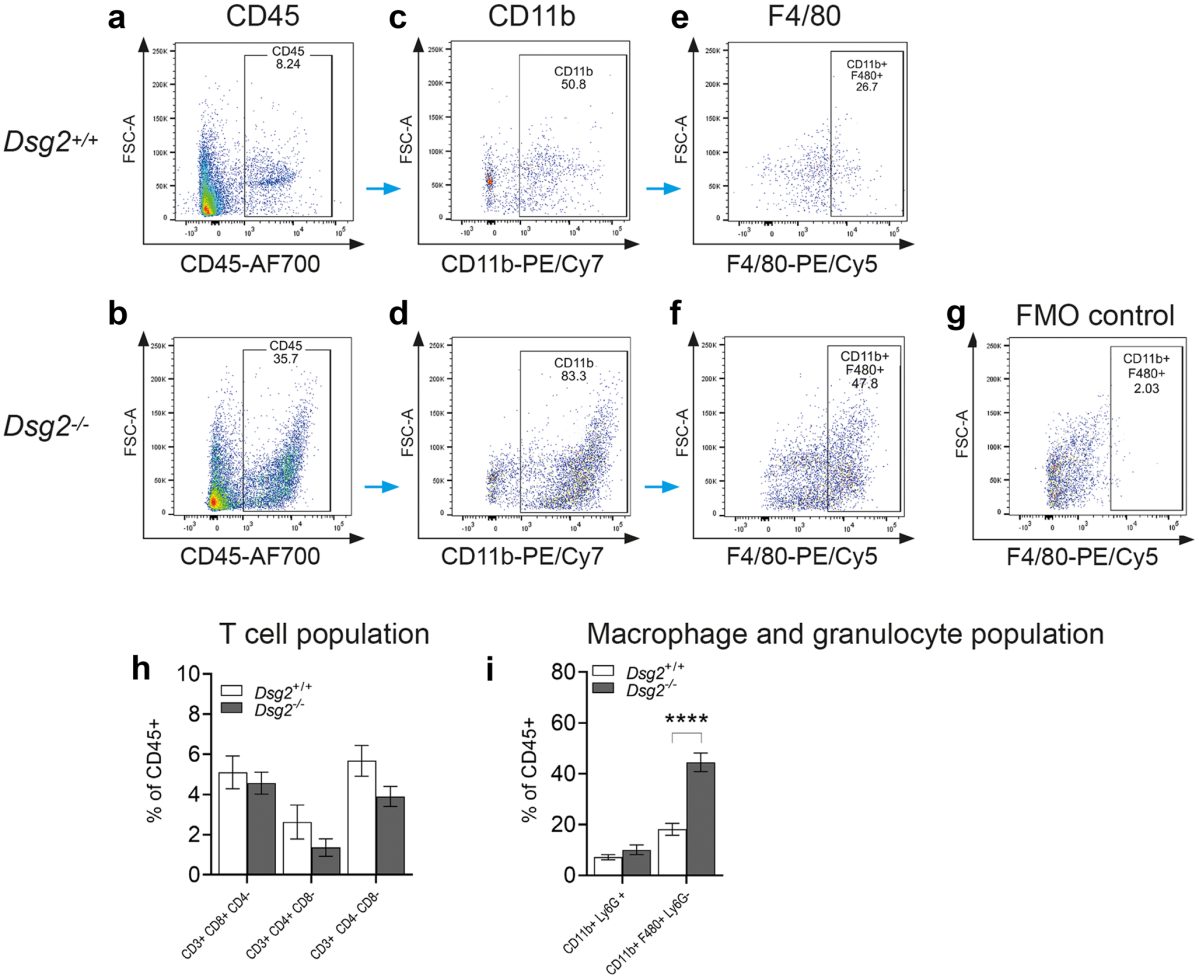
The unique inflammatory signature observed in the *Dsg2*^*−/−*^ postnatal heart contains a large macrophage population. (**a**–**g**) Example plots of the gating strategies used to identify macrophages with CD45 (**a**, **b**), CD11b (**c**, **d**) and F4/80 (**e**, **f**) antibodies in 2-week-old *Dsg2*^+/+^ and *Dsg2*^−/−^ hearts. The FMO control is shown in (**g**). The *Dsg2*-null heart contains a large population of CD45 + cells that are predominately macrophages (**b**, **d**, **f**). (**h**, **i**) Detailed FACS analysis of CD45 + cell population at 2 weeks in *Dsg2*^+/+^ and *Dsg2*^−/−^ hearts. Bar graphs show the distribution of CD45 + cells; cells were either CD3 + T cells populations (**h**) or CD3 − macrophage or granulocyte population (**i**). There is a large macrophage population in *Dsg2*^*−/−*^ hearts when compared to their littermate controls. Data represent mean ± SEM. ****p < 0.0001

Histological, immunohistochemical and RNA-Seq data suggests the presence of inflammatory infiltrate in 2-week *Dsg2*^*−/−*^ hearts. Therefore, a heat map was generated from 41 differentially expressed immune-related genes (c). One gene of interest included a significant increase in the inflammatory cell marker *CD45* (*Ptprc*), and further analysis of the transcriptome data revealed that the main population cells within the lesion appeared to be predominately macrophages. Upregulated genes associated with activated and mature macrophages *Adgre1* (or its common name *F4/80*), *Mpeg1*, *Fcgr1* and *Cd68* (d, d′, d′′ and d′′′). *Mpeg1* is also listed as one of the top 15 genes in the 2-week dataset (Table 3). Cells of monocyte lineage express CD68, a protein highly expressed by monocytic phagocytes, macrophages and osteoclasts. This gene is also expressed by circulating and tissue macrophages. Toll-like receptor protein 2 (*Tlr2* or *Cd282*) and *Tlr9* (*Cd289*) both recognise foreign material and can initiate any innate immune activation pattern including a macrophage-mediated response (d′′′′ and d′′′′′). The data in Table 5 lists additional M1 and M2 macrophage markers, cytokines and factors that are also upregulated in the 2-week *Dsg2*^*−/−*^ RNA-Seq dataset. Our data show both M1 and M2 genes are upregulated. We were also able to identify mouse-specific M2 cell surface markers such as *Fizz1*, *Ym1/2* and *Arg1* (Raes et al. 2002). We acknowledge that the M1/M2 paradigm of macrophage polarisation is a complex process and gene expression profiles will vary in different cardiac diseases or injury models (Nahrendorf and Swirski 2016). It is evident that distinct pathways are activated in the *Dsg2*-null postnatal and adult hearts. Collectively, our findings show that the processes of inflammation and fibrosis are interconnected and further investigation into this relationship would unravel the complex phases of murine AC disease progression.

**Table 5:** Phenotype and function of M1 and M2 macrophage subsets identified with our transcriptome dataset (2 weeks versus 10 weeks). The table lists a range of macrophage genes that were significantly upregulated in the *Dsg2*^*−/−*^ mouse heart at 2 weeks

	Macrophage subtype	
	M1	M2
Inducer	*Ifnγ*	*Tgfβ1*, *Il6*, *Il-1R*
Cell marker	*Cd86*, *Tlr2*, *Tl4*	*Tlr1*, *Tlr8*, *Cd163*, *Cd206* Mouse only: *Fizz1*, *Ym1/2*, *Arg1*
Cytokine	*Tnfα*, *Il-6*, *Il1R*	*Tgfβ1*, *Il-6*
Chemokine	*Ccl2*, *Ccl3*, *Ccl4*, *Ccl5*, *Ccl8*, *Ccl9*, *Ccl11*	*Ccl2*, *Ccl17*, *Ccl22*, *Ccl24*
Function	Pro-inflammatory, involved in phagocytosis	Anti-inflammatory, involved in tissue repair

Abbreviations for upregulated genes in the table: *Ccl* chemokine (C–C motif) ligand, *CD* cluster of differentiation, *Ifn-γ* interferon-gamma, *Il* interleukin, *MHC* major histocompatibility complex, *Tlr* Toll-like receptor, *Tnf-α* tumour necrosis factor alpha, *Tgf-β* transforming growth factor beta. Mouse-specific genes: *Ym1/2* chitinase 3-like 3, *Fizz1* resistin-like molecule α1, *Arg* arginase

### The inflammatory infiltrate observed at 2 weeks consists of macrophages

To further characterise the cellular infiltrate observed in the lesional areas, DAB chromogenic staining for inflammatory cells with CD45 and F4/80 for macrophages was performed (Fig. 4). *Dsg2*^+*/*+^ hearts showed no inflammatory cells within the myocardium with the majority located within the blood vessel (a and a′′). However, in the myocardium of cardiac-specific *Dsg2*^−/−^ mice, there was an increase in inflammatory cell marker CD45 staining in the lesional areas (previously described in Fig. 2), particularly within the epicardial surface and intraventricular septum (a′ and a′′′). The DAB staining also confirmed the presence of macrophages (F4/80) in the *Dsg2*^*−/−*^ heart (a′′′′′), but these were absent in the littermate control (a′′′′). The increase in CD45 + cells in 2-week-old *Dsg2*^*−/−*^ hearts when compared to adult hearts (10 weeks) was confirmed by RT-PCR (b) and RNA-Seq analyses (b′). Increased expression of chemokine monocyte chemotactic protein MCP-1 (*Ccl2*) and its receptor C–C motif chemokine receptor 2 (*Ccr2*) in the 2-week *Dsg2*^*−/−*^ heart suggests macrophage recruitment is initiated during early cardiac development to clear damaged/stressed cardiomyocytes (c, c′, c′′ and c′′′) (Mosser and Edwards 2008).

To shed more insight into these results, we examined the inflammatory infiltrate observed at 2 weeks with FACS analysis (Fig. 5). Specific gating strategies were employed to isolate and identify the immune cell population from whole hearts (a–g). Cells were isolated with CD45, CD11b and F4/80 antibodies (n = 7, both groups). There was a significant increase in CD45 + cells in the *Dsg2*^*−/−*^ heart (a and b) when compared to their littermate controls and a specifically large CD11b-positive population (c and d) that includes cells of the innate immune response such as neutrophils, monocytes, granulocytes and macrophages. Earlier transcriptome analysis suggested the presence of macrophages in the young diseased heart we gated with F4/80, which confirmed that a high proportion of these CD45 + cells were macrophages (e and f). To examine whether a T cell population was within the CD45 + cells, CD3, CD4 and CD8 antibodies were used, which revealed a relatively low number of T cells isolated from both groups (h and i).

### Investigation of cardiac cell death

**Fig. 6:**
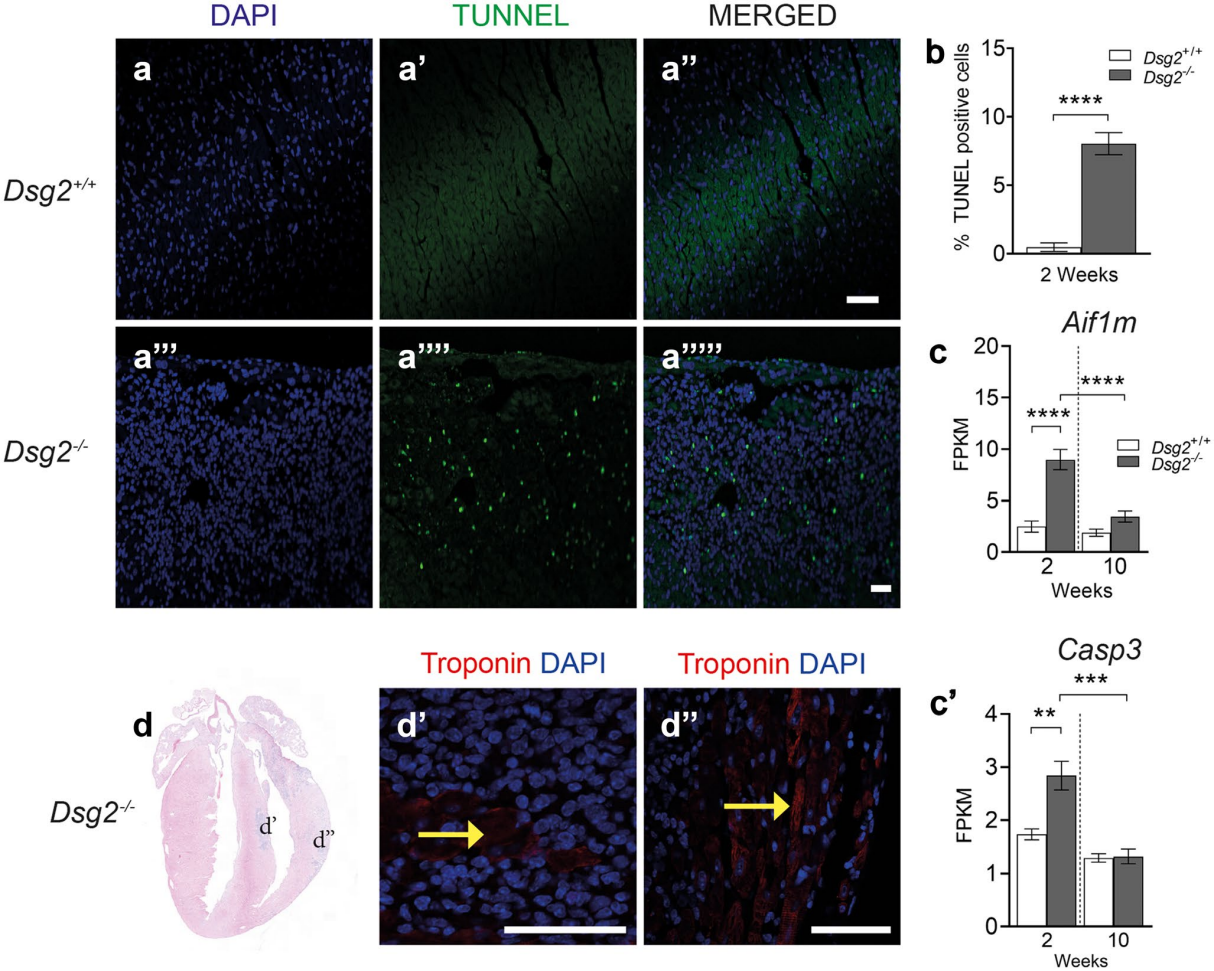
Apoptosis and inflammation occur simultaneously in the diseased postnatal heart. (**a**–**a′′′′′**) TUNEL analysis in 2-week-old *D*sg2^+/+^ (**a**–**a′′**) and *Dsg2*^*−/−*^ (**a′′′**–**a′′′′′**) hearts; scale bar represents 50 µm. (**b**) Bar graph to show there is a higher TUNEL index in the *Dsg2*^−/−^ hearts when compared to their control littermates. (**c**, **c′**) RNA-Seq analysis reveals the mitochondrial genes apoptosis-inducing factor 1 *Aif1m* (**c**) and caspase 3 *Casp3* (**c′**) were both upregulated at 2 weeks, suggesting nuclear disassembly during apoptosis. All data represent mean ± SEM. **p < 0.01, ***p < 0.001 and ****p < 0.0001. (**d**–**d′′**) Immunohistochemistry of two different lesional areas in 2-week-old *Dsg2*^*−/−*^ heart. These examples show the dominance of the inflammatory infiltrate (**d′**) and evidence of myocardial disarray (**d′′**). Scale bar represents 50 µm (**d′**, **d′′**)

To assess cell death in 2-week-old *Dsg2*^*−/−*^ hearts, TUNEL was used to quantify apoptosis rates (Fig. 6). The results showed higher apoptosis rates in the *Dsg2*^*−/−*^ group at 8% compared to 0.47% in the *Dsg2*^+/+^ group (a, a′, a′′, a′′′, a′′′′, a′′′′′ and b). The RNA-Seq analysis revealed mitochondrial apoptosis-inducing factor 1 (*Aifm1*), which activates chromatin condensation and DNA fragmentation, and caspase 3, a crucial mediator of apoptosis, both upregulated in the 2-week-old *Dsg2*^*−/−*^ heart (c and c′′). Immunohistochemistry showed few cardiomyocytes (assessed by troponin T positivity) in the lesional areas of the *Dsg2*^*−/−*^ heart, suggesting that cardiomyocytes are surrounded by macrophages (packed nuclei), most likely linked to phagocytosis (d, d′ and d′′). Numerous attempts were made with commercial kits to quantify necrosis in freshly isolated cardiomyocytes from *Dsg2*^*−/−*^ hearts. However, due to the proximity of the fibrotic areas and smaller sized hearts from these younger animals, isolating sufficient intact cardiomyocytes for this assay proved challenging. We recognise that this is a limitation of our study. However, electron micrograph analysis from 2-week *Dsg2*^*−/−*^ murine hearts exhibit swollen mitochondria, crista disarrangement and empty mitochondria/vacuoles consistent with apoptosis and necrosis (Supporting Fig. 4). These results suggest that loss of *Dsg2* causes cardiac stress and a combination of cardiomyocytes, dying macrophages and cardiac interstitial cells undergo apoptosis around the lesions to facilitate disease progression.

### iRhom2/ADAM17 activation in the 2-week Dsg2^−/−^ heart

**Fig. 7:**
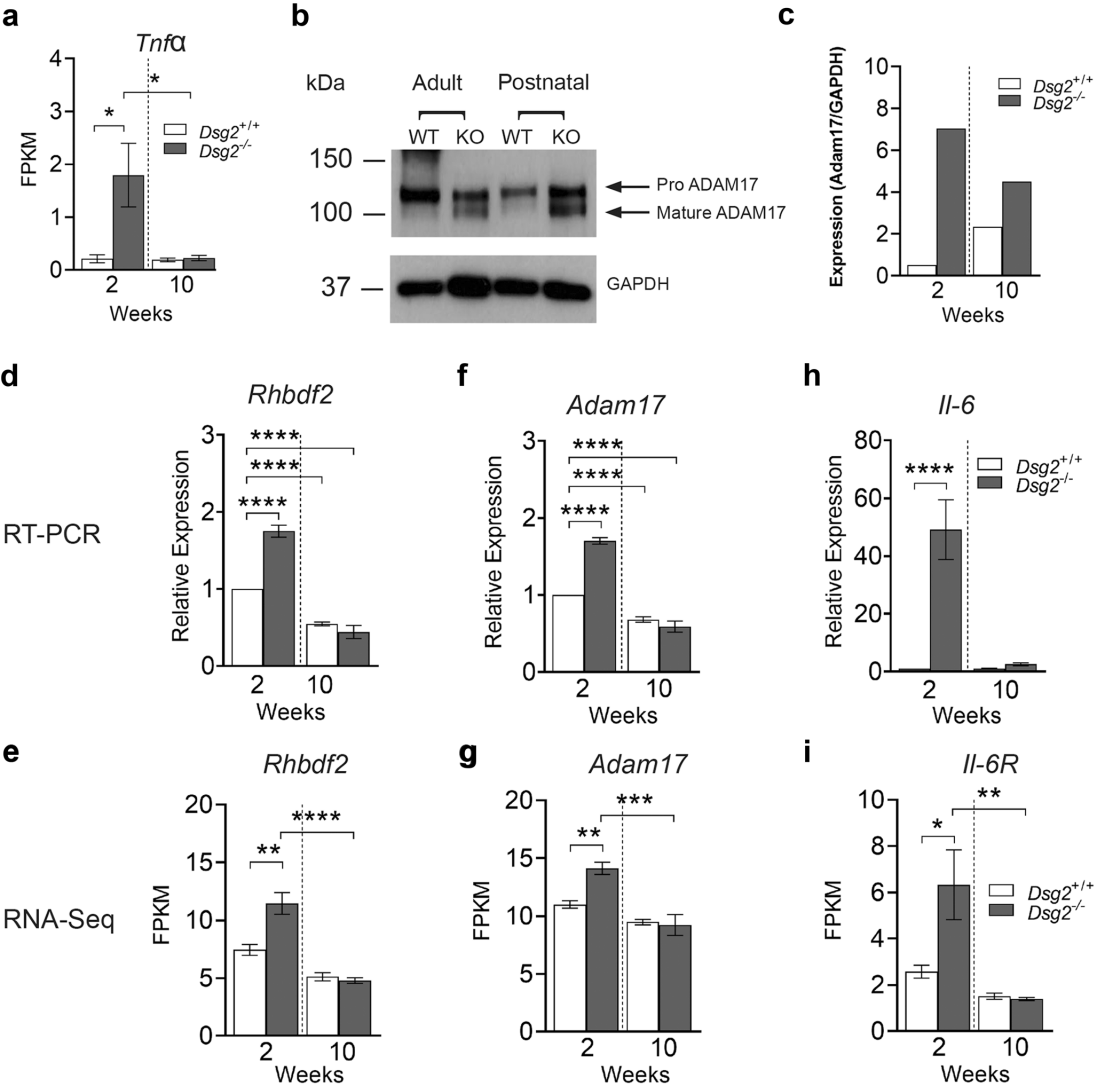
Transcriptome data reveals early activation of stress and inflammatory-associated pathways in 2-week-old *Dsg2*^−/−^ hearts when compared to 10-week-old adult cohort. (**a**) The cytokine *Tnfα* is highly expressed at this early stage of development. (**b**, **c**) The iRhom2/ADAM17 pathway is upregulated in the 2-week-old *Dsg2*^−/−^ heart. Western blotting of lysates from adult and postnatal mouse heart with GAPDH as loading control. Densitometric analysis reveals increased mature ADAM17 (~ 93 kDa) in the 2-week-old *Dsg2*^*−/−*^ heart (**b**). Each positive band was normalised to GAPDH and quantified by NIH ImageJ (**c**). Data represent results from a single experiment. (**d**–**i**) Quantitative PCR analysis and RNA-Seq confirm the activation of *Rhbdf2* (**d**, **e**) and *Adam17* (**f**, **g**) at this early time point. Pro-inflammatory cytokine IL-6 (**h**) is also highly expressed at this stage; IL-6R (**i**), a substrate of Adam17, was also detected with RNA-Seq data. All bar graphs represent mean ± SEM. *p < 0.05, **p < 0.01, ***p < 0.001 and ****p < 0.0001

One pathway of interest upregulated in the 2-week *Dsg2*^*−/−*^ mouse is the iRhom2/ADAM17 stress pathway (Fig. 7). KEGG pathway analysis initially showed the TNF signalling pathway was upregulated in the 2-week *Dsg2*^*−/−*^ cohort. On closer inspection, RNA-Seq revealed the cytokine tumour necrosis factor alpha (Tnfα) was significantly elevated at 2 weeks in the *Dsg2*^−/−^ mouse heart (a). TNFα is cleaved from the cell surface by ADAM17 (or TNFα-converting enzyme, TACE), an ectodomain sheddase enzyme. Mature Adam17 promotes the specific cleavage of a number of other ligands such as Il-6r and *Dsg2*. Western blot analysis revealed elevated levels of mature Adam17 in 2-week-old *Dsg2*^*−/−*^ heart, indicating increased sheddase activity (b and c). RT-qPCR and RNA-Seq showed increased levels of *Rhbdf2* (d and e) and *Adam17* (f and g) in the 2-week-old *Dsg2*^*−/−*^ heart. *Rhbdf2* encodes the inactive Rhomboid 2 (iRhom2) that regulates and promotes the maturation of ADAM17 (Adrain et al. 2012; McIlwain et al. 2012). The increase in *Il-6*, the ligand for Il-6r, was also identified by qRT-PCR, and RNA-Seq showed increased IL6r (h and i). Il-6 may be released directly from macrophages and/or stressed cardiomyocytes, facilitating changes in the myocardium from a physiological to pathological state.

## Discussion

**Fig. 8:**
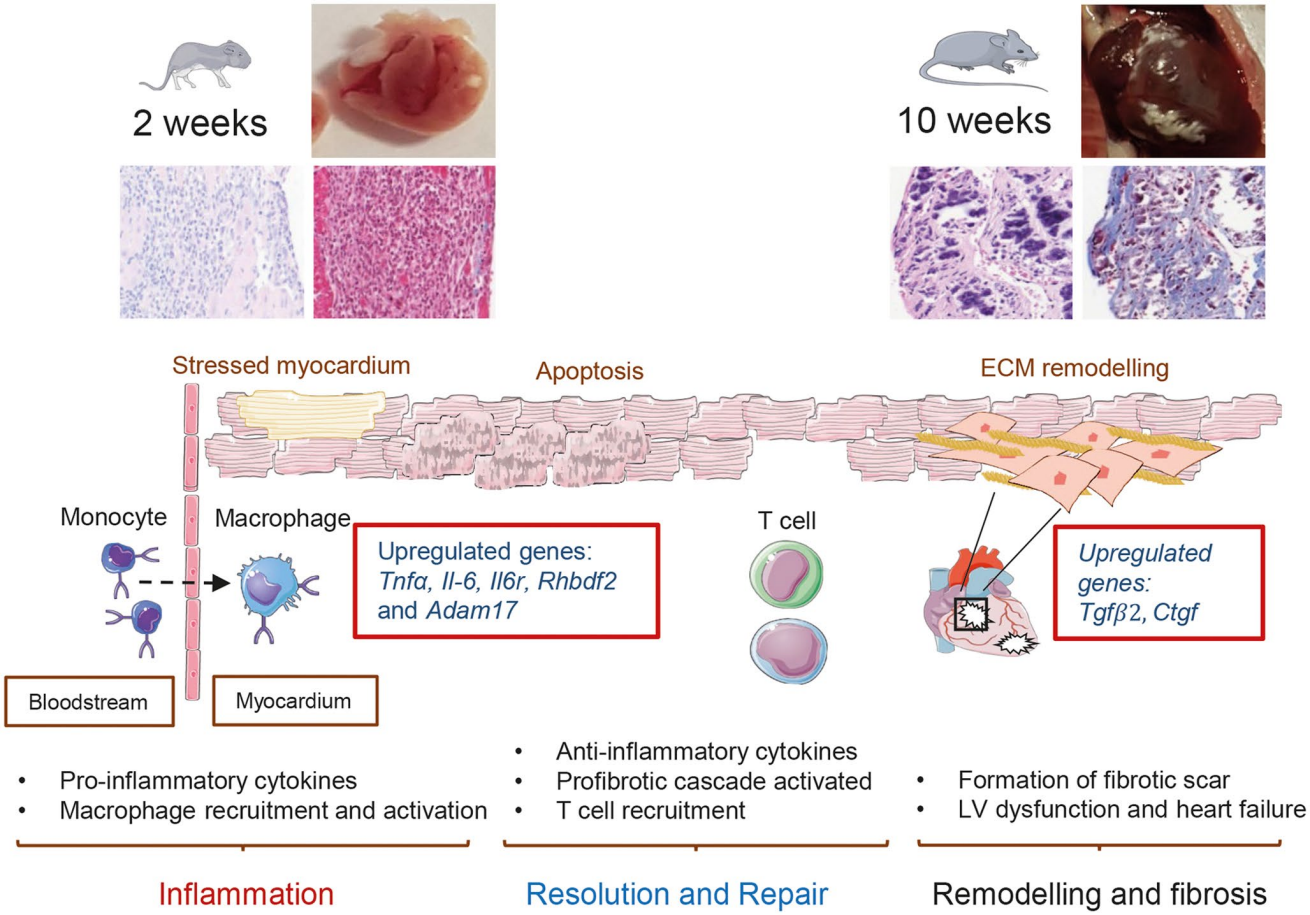
Summary of the proposed key events in cardiac-specific murine model of AC. The loss of desmoglein 2 can trigger cell death and initiate the immune response. Postnatal *Dsg2*^−/−^ hearts (2 weeks) suggest inflammation occurs excessively in response to desmosome dysfunction. Our findings reveal key genes are upregulated during the early innate immune response. Pro-fibrotic genes are also expressed at this postnatal period resulting in the pathological condition observed in *Dsg2*^*−/−*^ adult mice. (This figure was created with templates from Servier Medical Art, licensed under a Creative Common Attribution 3.0 Generic License. http://smart.servier.com)

To summarise our findings, the inflammatory response and secretion of pro-inflammatory cytokines we identified may play an important role in generating the phenotype in the desmoglein 2 cardiac-specific mouse model of AC. Macrophage recruitment and activation follow and are accompanied by the activation of the fibrotic pathways, eventually leading to a scarred poorly functional ventricle (Fig. 8). Transcriptome data and cellular phenotyping suggest key genes that may drive the inflammatory process and profibrotic cascade which lead to severe cardiomyopathy. The data support early inflammatory gene expression at 2 weeks followed by a more fibrotic picture later at 10 weeks. These multiple sequential events are likely to mould the disease phenotype caused by loss of desmoglein 2. We were able to demonstrate consequent apoptosis which includes but is not exclusive to myocytes. However, there were technical issues with assays for necrosis though it was likely to be present. Previously, the cardiac-specific N271S-*dsg2* mouse model also showed inflammatory infiltrates in the myocardium of 2–3-week-old mice and found both necrosis and apoptosis occurred in the early stages of cardiac development (Pilichou et al. 2009).

The potential role of inflammation as a potential disease driver rather than a bystander activated as an epiphenomenon is only beginning to be appreciated (Austin et al. 2019; Chelko et al. 2019). In agreement with our study, a recent report has also shown that inflammation does shape the pathogenesis of murine AC (Lubos et al. 2020). The presence of inflammatory cell infiltration in human AC cases has been known for some time (Basso et al. 1996), but it was uncertain whether these inflammatory events lead to disease pathogenesis or are an epiphenomenon due to cardiomyocyte injury. Interestingly, cardiac inflammation was present in 60–80% of AC. Later studies also described signs of distinct inflammatory infiltrates in postmortem biopsies from patients diagnosed with AC (Campuzano et al. 2012). Interestingly, six AC paediatric patients were recently identified with specific desmosomal mutations (Martins et al. 2018). Cardiac magnetic resonance imaging confirmed myocardial inflammation, and these inflammatory events were correlated with severe reduced biventricular cardiac function (Martins et al. 2018; Patrianakos et al. 2012). There are phenotypic similarities in these young patients with lesions present in our 2-week *Dsg2*^*−/−*^ mouse hearts. The viral trigger was not responsible for myocarditis-like episodes, but half of the cases were exercise induced. These ‘hot phases’ are often associated with a rise in troponin levels and cardiac inflammation. It is widely accepted that the AC phenotype may be worsened by stressors such as exercise and the clinical course can be punctuated by acute arrhythmic episodes (Sen-Chowdhry et al. 2010). Inflammatory genes may also be considered as potential markers in the early stages of disease management in AC families. The recent examination of 42 human AC probands showed the association of circulating autoantibodies against cardiac and intercalated disc proteins with AC disease development (Caforio et al. 2020). Studies in human and boxer dogs showed anti-DSG2 autoantibodies were identified as a specific marker of AC (Chatterjee et al. 2018; Koga et al. 2013). This will be an interesting area for future work in murine models.

The early immune response at 2 weeks in *Dsg2*^−/−^ mice was characterised by substantial macrophage infiltration including increased expression of genes associated with pro-inflammatory M1 macrophages (Chávez-Galán et al. 2015; Liu et al. 2014). M1 activated macrophages secrete pro-inflammatory cytokines such as IL-1, IL-6 and TNFα, and these and related components in the pathways were increased in expression in our data. In addition to this, stressed cardiomyocytes can release TNFα to trigger the inflammatory process and activate the immune cell population to clear dying cells via apoptosis or necrosis (Dobaczewski et al. 2011). The increased expression of genes such as *Ccl2* and *Ccr2* emphasises the important role of tissue macrophages in damaged *Dsg2*^*−/−*^ hearts. These genes are active during the innate immune response that controls the migration and infiltration of monocytes/macrophages. However, it is important to note that the M1/M2 paradigm is increasingly challenged as expression profiles can vary in different disease models (Nahrendorf and Swirski 2016). Several genes that are indicative of an M2 macrophage phenotype (and, consequently, type 2 immunity) were upregulated in our bulk RNA-sequencing dataset in 2-week *Dsg2*^−/−^ hearts. The early inflammatory process is also accompanied by the upregulation of the iRhom2/Adam17 stress pathway and its downstream targets such as Il-6. We observed an increase in Il-6R and Tnfα in 2-week *Dsg2*^*−/−*^ hearts with RNA-Seq and RT-PCR; the cytokine profile suggested the role of Adam17 sheddase activity. Mature Adam17 was only detected in the *Dsg2*^*−/−*^ hearts.

We originally gathered information from our cardiac-specific desmoglein 2 mouse model by first comparing the echocardiographic and histological data in the *Dsg2* heterozygous knockout mouse (*Dsg2*^+*/−*^) with control (*Dsg2*^+*/*+^) and knockout (*Dsg2*^*−/−*^) cohorts. The decline in cardiac function seen in the homozygotes was comparable to that seen in other models (Krusche et al. 2011; Pilichou et al. 2009). The *Dsg2*^+*/−*^ cohorts would be equivalent to human studies as AC patients have heterozygous mutations for DSG2. The histological analysis revealed *Dsg2* haploinsufficiency did not cause inflammation or display distinct fibrous lesions in 10-week-old *Dsg2*^+*/−*^ hearts in the absence of additional provocation. Interestingly, echocardiographic analysis revealed these mice do exhibit a slight decrease in cardiac function, though not significant when compared to their wild-type littermates. In humans, a number of disease-causing heterozygous mutations in DSG2 are missense mutations and may have a dominant negative effect on the assembling desmosome. It is important to note that DSG2 mutations in humans are often more pathogenic in the heterozygous state when triggers such as exercise and stress are involved.

It is known that the heart contains a resident population of macrophages, and it is thought, that as in other tissues, they have a housekeeping function detecting potential myocardial injury and clearing cellular waste (Heidt et al. 2014). In addition, they may also have novel roles such as such as influencing excitability in the atrioventricular node (Hulsmans et al. 2017). The modest electrophysiological phenotype observed in this model is also indicative that arrhythmia may predominantly arise from fibrosis and scar formation rather than pronounced changes in conduction as seen with desmoplakin mutations (Gomes et al. 2012). The best characterised injury model is that of myocardial infarction, and there is a carefully sequenced process of acute innate inflammation driven largely by macrophages, which ultimately results in repair and scar formation (Swirski and Nahrendorf 2018) (Frodermann and Nahrendorf 2018; Nahrendorf 2018). Whilst there are some similarities, there are also differences. In our case, the two processes of inflammation and repair seem to occur simultaneously but largely terminate at 10 weeks. It is unclear if this is due to extensive myocardial damage or whether desmosomal disruption early in life as the heart matures postnatally is the critical driver.

Our observation that inflammation is most active early on suggests that intervention with immune-suppressing drugs should be given before extensive fibrosis sets in. IL-6 is implicated in chronic inflammation and cancer and is one potential therapeutic target for intervention (López-Mejías and González-Gay 2019). Glycogen synthase kinase-3 beta (GSK-β), a key player in the pathogenesis of AC, can also regulate the inflammatory response (Chelko et al. 2016). In addition, the success of small molecule inhibition of nuclear factor kappa B signalling implicates inflammatory processes (Chelko et al. 2019). Secondly, any clinical immune signature may be detected prior to overt disease onset and there may be benefits of therapeutic intervention at this early stage. Future studies in murine models of AC could explore further the use of known inhibitors of inflammation and fibrosis.

## Supplementary Information

Below is the link to the electronic supplementary material.

Supplementary file1 (TIF 11540 KB)

Supplementary file2 (TIF 11926 KB)

Supplementary file3 (TIF 7509 KB)

Supplementary file4 (TIF 11133 KB)

Supplementary file5 (TIF 4541 KB)
