# Supplementary figures and images for: Early inflammation precedes cardiac fibrosis and heart failure in desmoglein 2 murine model of arrhythmogenic cardiomyopathy
Source: Cell Tissue Res. 2021 Jul 8;386(1):79–98. doi: 10.1007/s00441-021-03488-7 (PMC8526453; doi:10.1007/s00441-021-03488-7)

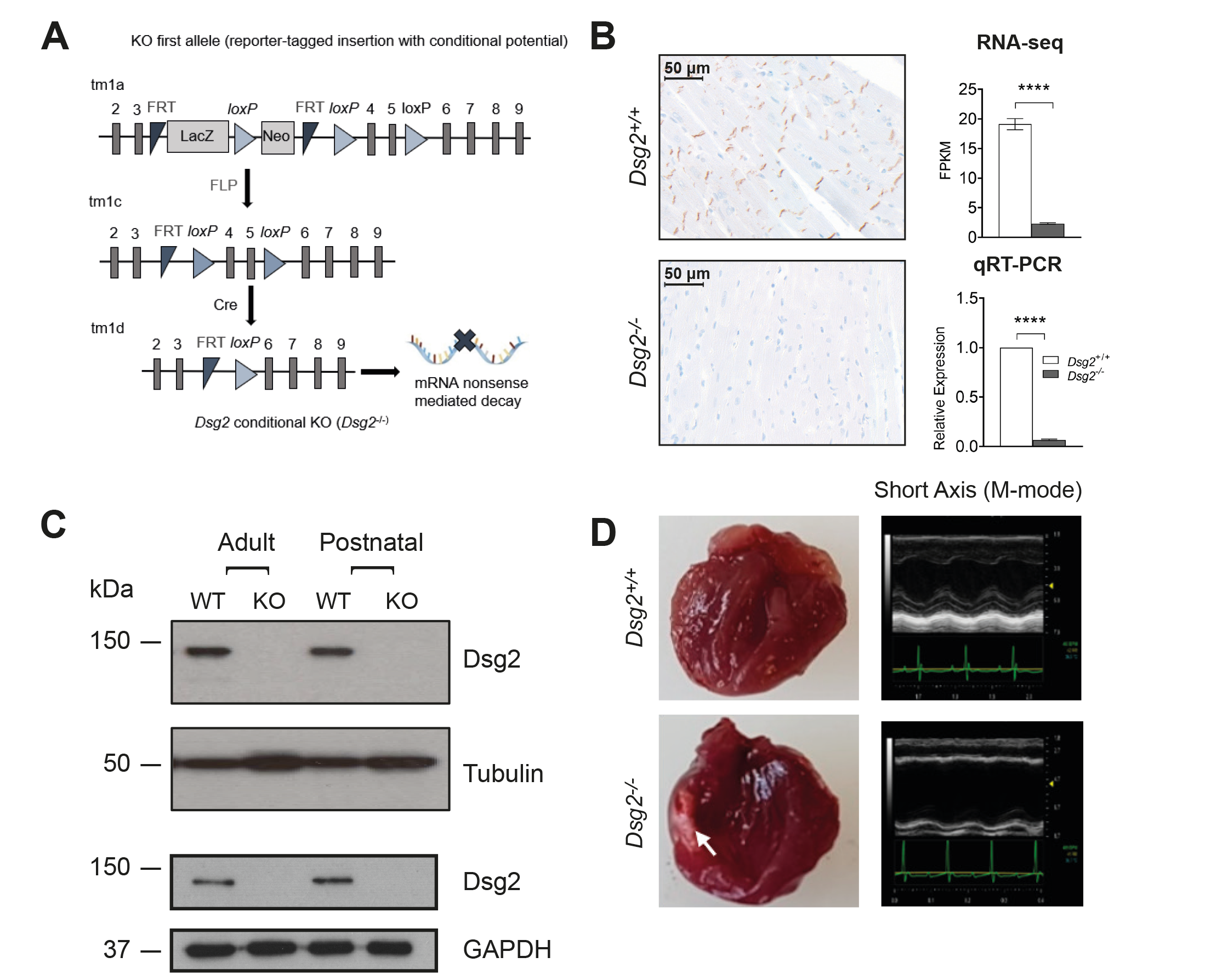

Supplement: Supplementary file 1 — Supplementary file1 (TIF 11540 KB) [file 441_2021_3488_MOESM1_ESM.tif]

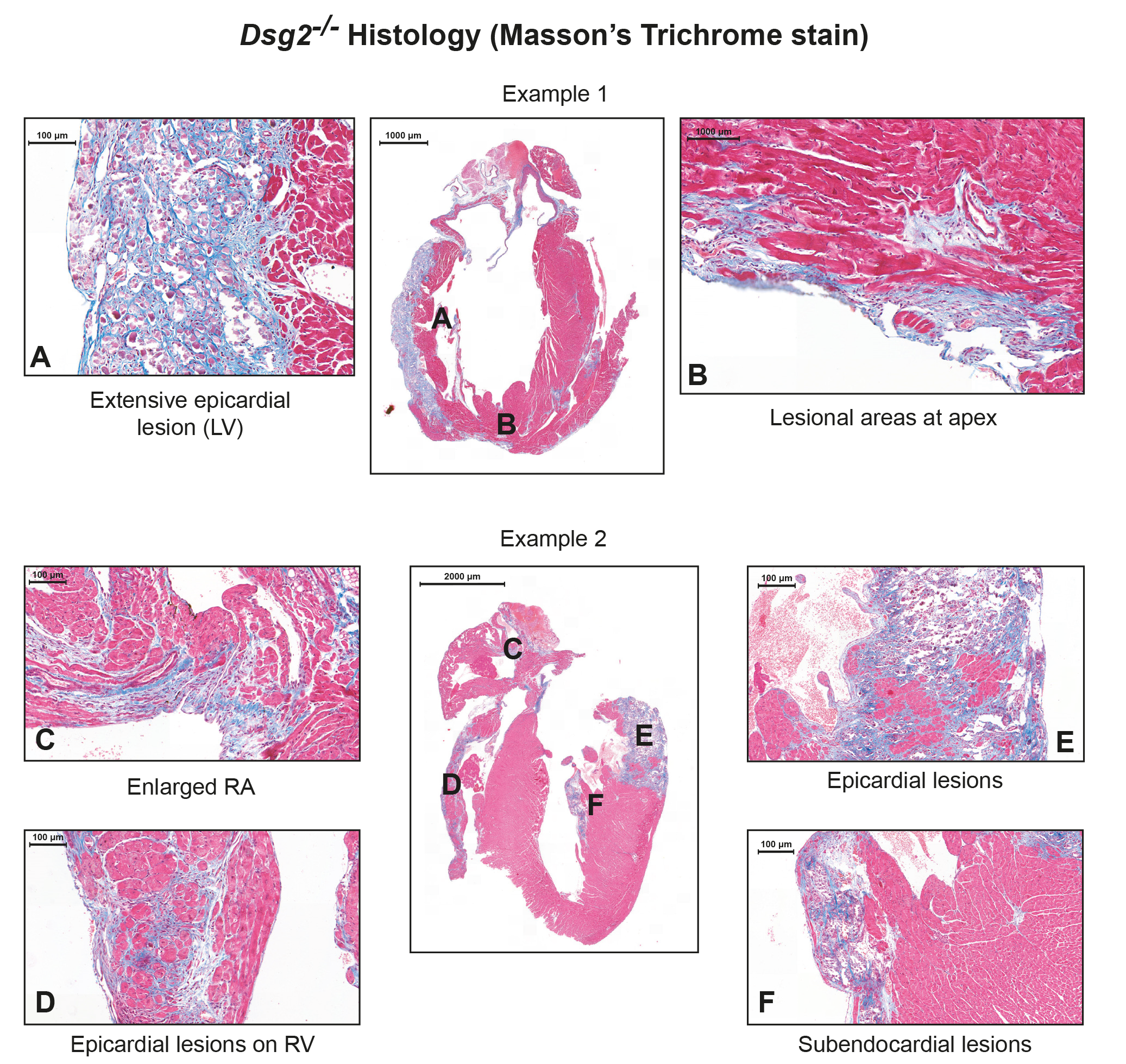

Supplement: Supplementary file 2 — Supplementary file2 (TIF 11926 KB) [file 441_2021_3488_MOESM2_ESM.tif]

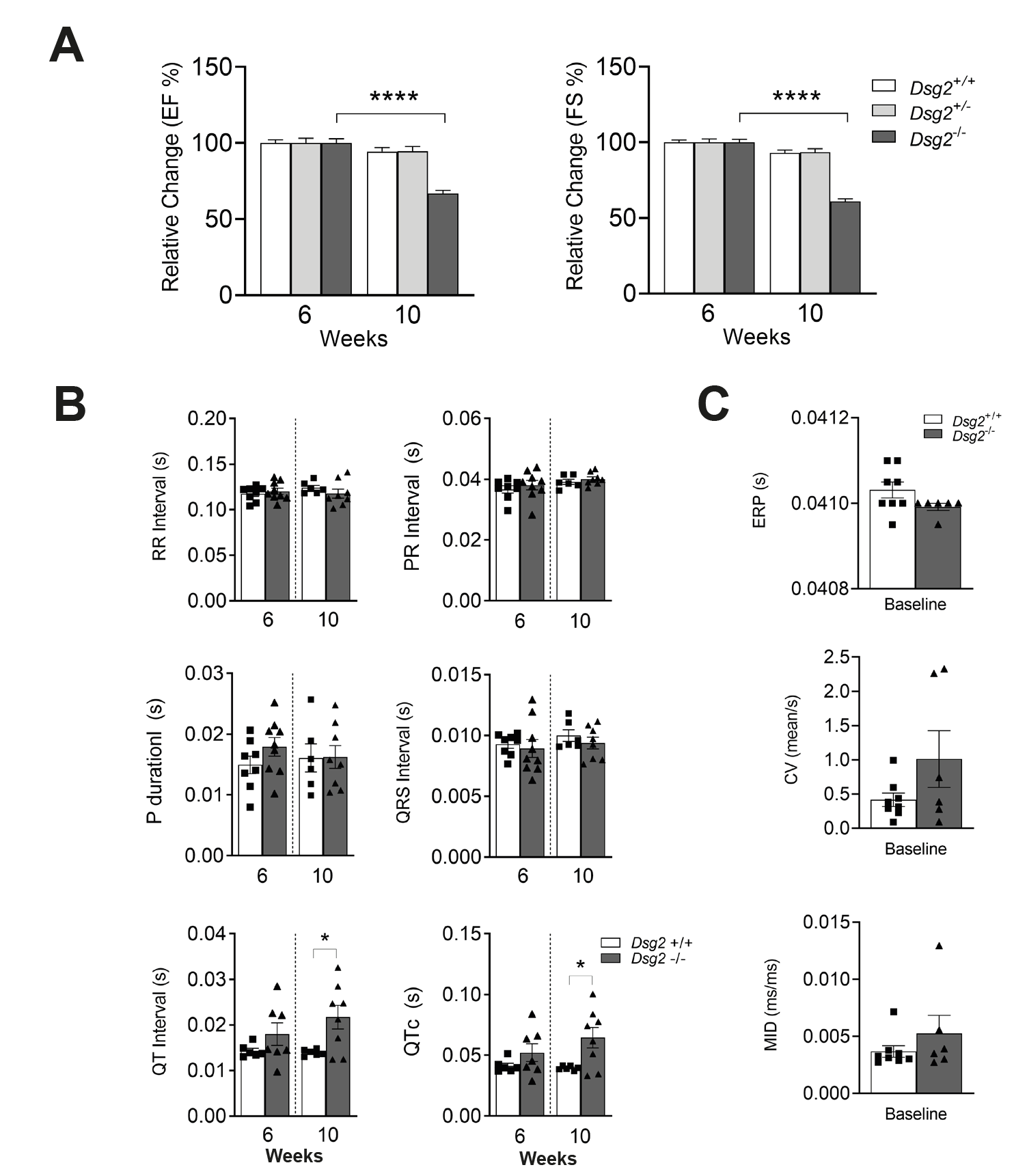

Supplement: Supplementary file 3 — Supplementary file3 (TIF 7509 KB) [file 441_2021_3488_MOESM3_ESM.tif]

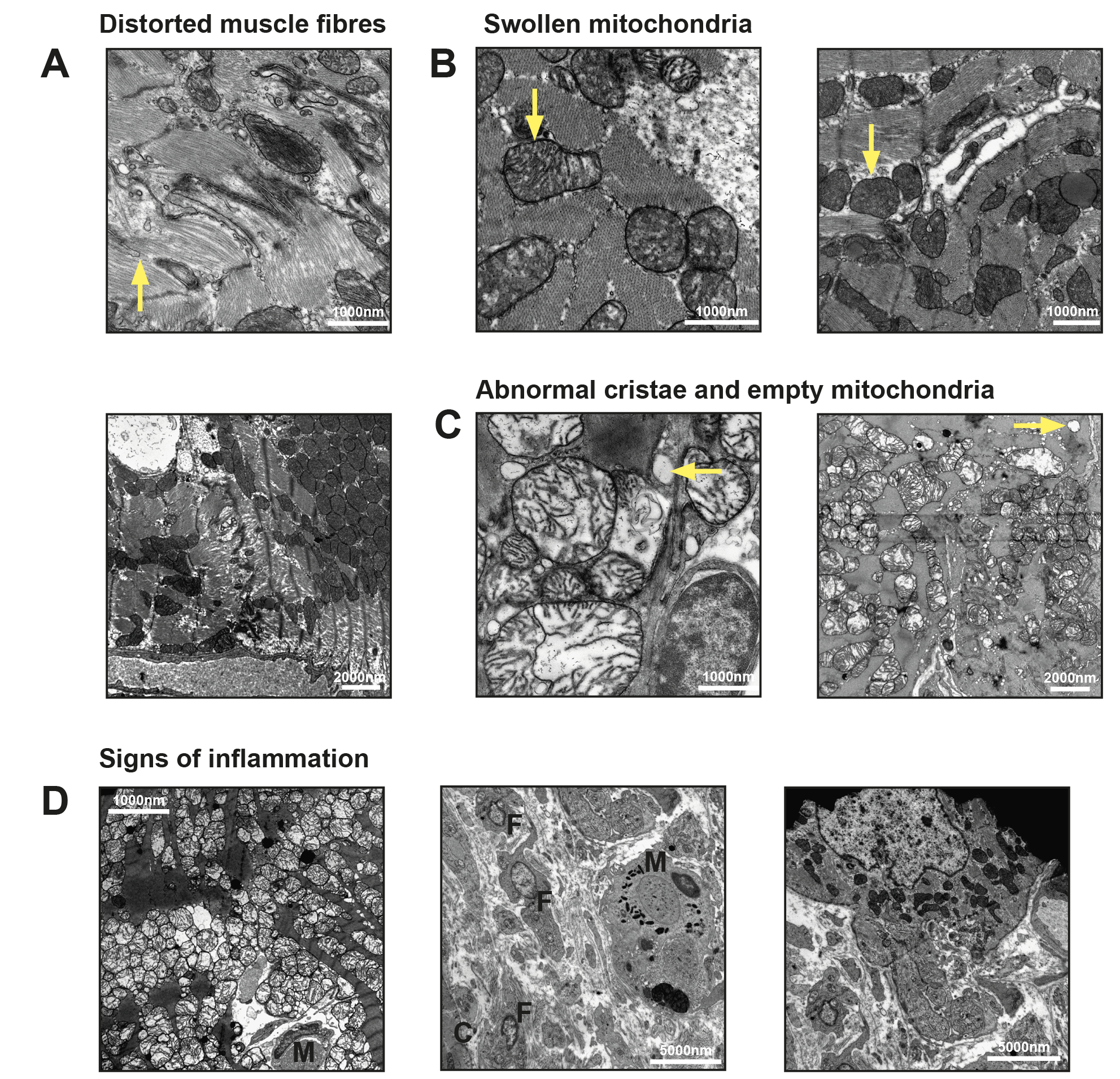

Supplement: Supplementary file 4 — Supplementary file4 (TIF 11133 KB) [file 441_2021_3488_MOESM4_ESM.tif]

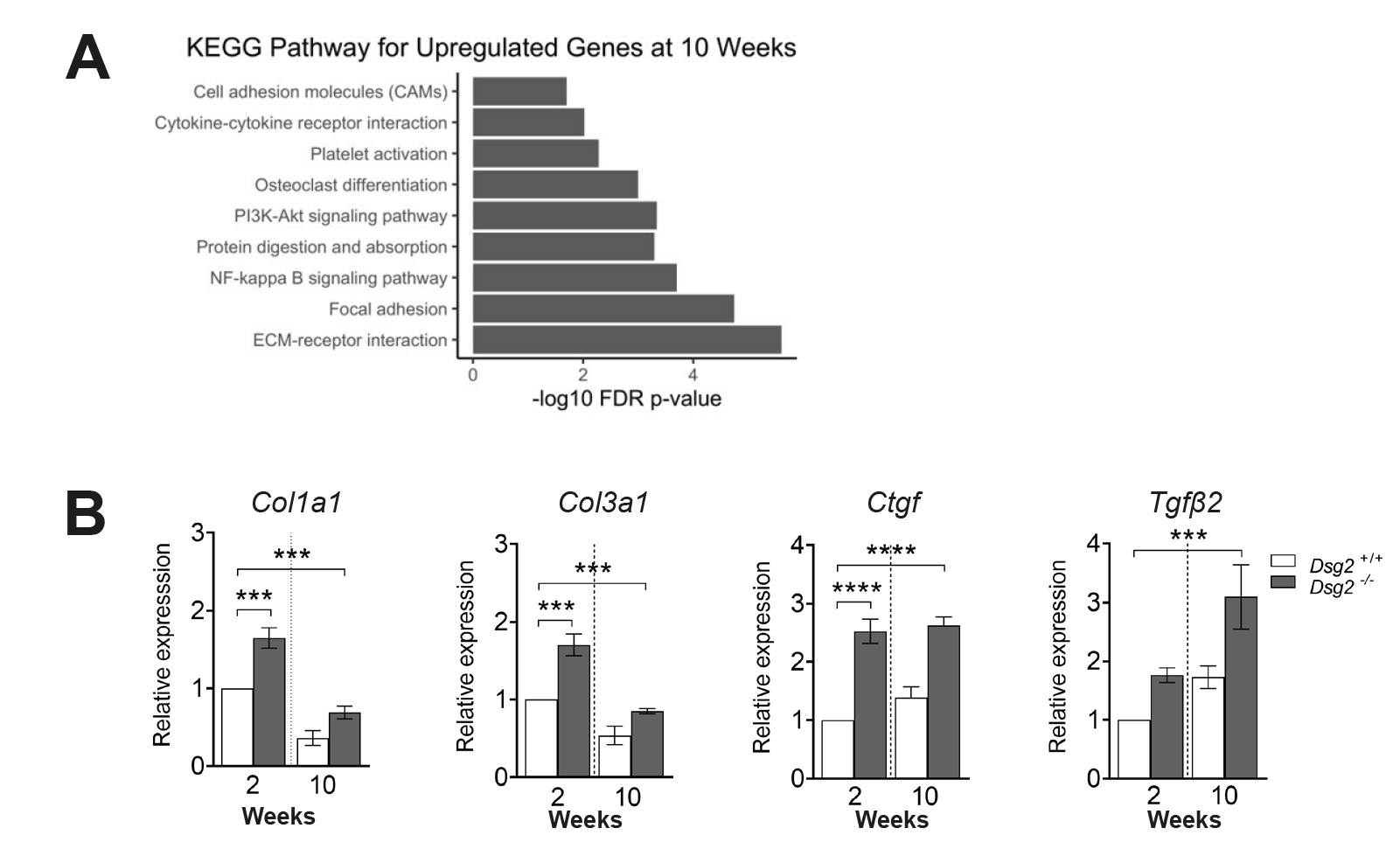

Supplement: Supplementary file 5 — Supplementary file5 (TIF 4541 KB) [file 441_2021_3488_MOESM5_ESM.tif]
